# Seleninic Acid Potassium Salts as Water-Soluble Biocatalysts with Enhanced Bioavailability

**DOI:** 10.3390/ma13030661

**Published:** 2020-02-02

**Authors:** Magdalena Obieziurska, Agata J. Pacuła, Anna Laskowska, Angelika Długosz-Pokorska, Anna Janecka, Jacek Ścianowski

**Affiliations:** 1 Department of Organic Chemistry, Faculty of Chemistry, Nicolaus Copernicus University, 7 Gagarin Street, 87-100 Torun, Poland; magdao@umk.pl (M.O.); pacula@umk.pl (A.J.P.); annlas@doktorant.umk.pl (A.L.); 2 Department of Biomolecular Chemistry, Faculty of Medicine, Medical University of Lodz, Mazowiecka 6/8, 92-215 Lodz, Poland; angelika.dlugosz@umed.lodz.pl (A.D.-P.); anna.janecka@umed.lodz.pl (A.J.)

**Keywords:** areneseleninic acids, areneseleninic acid salts, amphiphilic compounds, antioxidant activity, antiproliferative activity

## Abstract

Organoselenium compounds are well-known glutathione peroxidase (GPx) mimetics that possess antioxidants/prooxidant properties and are able to modulate the concentration of reactive oxygen species (ROS), preventing oxidative stress in normal cells or inducing ROS formation in cancer cells leading to apoptosis. The purpose of this study was the synthesis of potent GPx mimics with antioxidant and anticancer activity along with improved bioavailability, as a result of good solubility in protic solvents. As a result of our research, glutathione peroxidase (GPx) mimetics in the form of water-soluble benzeneseleninic acid salts were obtained. The procedure was based on the synthesis of 2-(N-alkylcarboxyamido)benzeneselenenic acids, through the oxidation of benzisoselenazol-3(2H)-ones or analogous arenediselenides with an amido group, which were further converted to corresponding potassium salts by the treatment with potassium tert-butanolate. All derivatives were tested as potential antioxidants and anticancer agents. The areneseleninic acid salts were significantly better peroxide scavengers than analogous acids and the well-known organoselenium antioxidant ebselen. The highest activity was observed for the 2-(N-ethylcarboxyamido)benzeneselenenic acid potassium salt. The strongest cytotoxic effect against breast cancer (MCF-7) and human promyelocytic leukemia (HL-60) cell lines was found for 2-(N-cyclohexylcarboxyamido)benzeneselenenic acid potassium salt and the 2-(N-ethylcarboxyamido)benzeneselenenic acid, respectively. The structure–activity correlations, including the differences in reactivity of benzeneseleninic acids and corresponding salts were evaluated.

## 1. Introduction

**Scheme 1:**
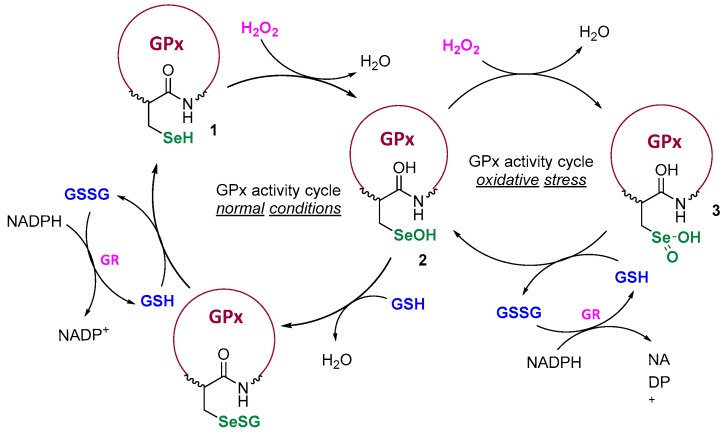
GPx activity cycle at physiological conditions and elevated ROS levels.

Designing catalysts inspired by enzymes is one of the crucial tactics aimed at influencing cell physiology or reversing a pathological state that triggers a disease to develop. The catalytic systems protecting cells from reactive oxygen/nitrogen species (ROS/RNS) and further oxidative damage, involving selenoenzymes from the glutathione peroxidase (GPx) family, can operate due to the presence of the reactive selenol moiety incorporated in the structure of the amino acid selenocysteine [1,2,3]. In the GPx-catalytic cycle, the initial redox-active -SeH, plays the crucial role at the enzyme’s active site and initiates the cycle through the rapid reaction with H_2_O_2_. As it has been presented by Mugesh et al., the regulation of ROS concentrations depends on the levels of peroxide and the thiol co-factor, mostly the glutathione (GSH) [4]. Under physiological conditions, the active selenol 1 is first oxidized to the selenenic acid 2 and next regenerated in the presence of two GHS molecules. In the state of oxidative stress, when the H_2_O_2_ level is higher, with a lower amount of thiol, the cycle is extended and further oxidation of the seleninic acid 3 takes place (Scheme 1).

**Scheme 2:**
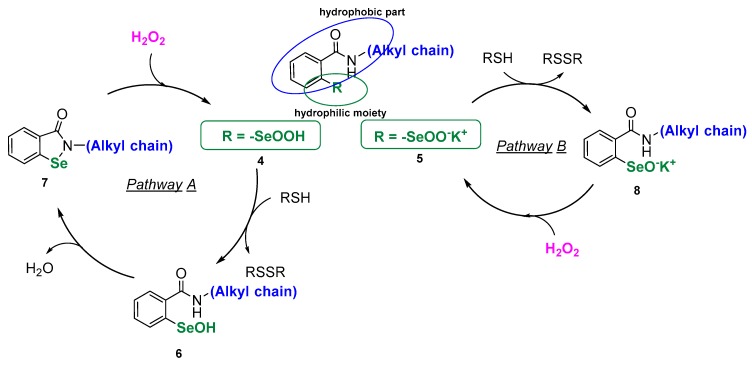
Origin of the structure of the designed antioxidants 4 and 5.

Since it was discovered that small organoselenium compounds can mimic the activity of glutathione peroxidase and reduce peroxides in a similar manner to the above-presented cycle, a lot has been accomplished in the synthesis of redox-active Se-catalysts [5]. Several compounds have been identified and selected as biologically potent, however, low selectivity, leading to higher toxicity, along with solubility problems are still limiting the applicability. When designing new GPx mimics in the form of modified “selenocysteine-like” catalysts, the direct approach to synthetize compounds bearing a free selenol moiety is problematic due to the high instability of the -SeH group and rapid formation of the corresponding diselenide. Thus, an efficient organoselenium antioxidant should possess a masked Se-moiety that could be easily unmasked and transformed to a reactive selenol at the specific place of action. This can be accomplished by synthetizing stable compounds that correspond structurally to a certain isoform of the GPx selenocysteinyl redox center. Herein, we present a series of new peroxide scavengers in the form of seleninic acids 4 and corresponding seleninic salts 5. Both of these Se(IV) compound series 4 and 5 can be qualified as the derivatives of the primal GPx in the form of seleninic acid 3. The structure of the designed molecules is composed of two important features: the hydrophilic -SeOOH/SeOOK group and the hydrophobic N-alkylbenzamide moiety that connects together exhibit amphiphilic properties. As the structure of derivative 4 corresponds to the specific benzseleninic acid intermediated formed in the GPx catalytic cycle of the known drug candidate ebselen, the mechanism of action should include the “ebselen-like” catalytic pathway A. However, we assume that in the case of analogue salt 5, the formation of the Se-N bond will not be achieved, which can accelerate the speed of peroxide reduction (pathway B, Scheme 2).

Amphiphilic molecules, that can easily penetrate lipid bilayer membranes, may create a delivery system with an incorporated pharmacophore. The Se(IV) molecules can act as “the hidden selenol”, as postulated by Rocha [1], be transported to the specific place of action as stable and soluble RSeOOH/RSeOOK and, at the final stage, be transformed into the bioactive RSeH.

Till now, seleninic acids have found several application routes, including their utilization as ligands [6,7,8,9,10] and reagents in oxidation [11,12,13,14], dehydrogenation [15] and oxidative deoximation reactions [16]. However, their applicability in medicinal chemistry seems to be still an unexplored field with promising perspectives. Only few examples of such areneseleninic acids have been published by Mugesh et al. [4,17,18,19], but analyzed only as intermediates of corresponding benzisoselenazolones, with no activity evaluation. Similar amphiphilic seleninic acids with *p*-amido function have been presented by Jacobs et al. [20]. The studies revealed that the compounds can easily penetrate cell membranes and possess significantly better antibacterial activity than phenyl seleninic acid and simple surfactants. Nevertheless, the “ebselen-like” catalysts have never been synthetized in the form of seleninic acid salts. The purpose of this study was the synthesis of GPx mimics in the form of RSeOO^-^M^+^. The presence of the -SeOO^−^M^+^ moiety can improve the solubility in water and change the physicochemical properties of the molecules which, in turn, may strongly influence the metabolism of the presented potential drug candidates.

## 2. Materials and Methods

### 2.1. General

Melting points were measured with a Büchi Tottoli SPM-20 heating unit (Büchi Labortechnik AG, Flawil, Switzerland) and were uncorrected. Nuclear magnetic resonance (NMR) spectra were recorded on Bruker Avance III/400 or Bruker Avance III/700 (Karlsruhe, Germany) for 1H and 176.1 MHz or 100.6 MHz for 13C. Chemical shifts were recorded relative to SiMe_4_ (δ0.00) or solvent resonance (CDCl_3_ δ7.26, CD_3_OD δ3.31). Multiplicities were given as: s (singlet), d (doublet), dd (double doublet), ddd (double double doublet), t (triplet), dt (double triplet), and m (multiplet). 77Se NMR spectra were recorded on Bruker Avance III/ 400 or Bruker Avance III/ 700 with diphenyl diselenide as an external standard. NMR spectra were carried out using ACD/NMR Processor Academic Edition. All original NMR spectra are presented in Supplementary Materials. Infrared spectra (IR) were measured on Alpha FT-IR spectrometer from Bruker (Karlsruhe, Germany). Elemental analyses were performed on a Vario MACRO CHN analyzer. Commercially available solvents dimethylformamide (DMF), dichloromethane (DCM), and MeOH (Aldrich, St. Louis, MO, USA) and chemicals were used without further purification. Column chromatography was performed using Merck 40-63D 60Å silica gel (Merck, Darmstadt, Germany). 

### 2.2. Procedures and Analysis Data

#### 2.2.1. Synthesis of 2-(N-alkylcarboxyamido)benzeneselenenic acids 10–15

Method A: To a solution of N-alkylbenzisoselenazol-3(2H)-one (1.00 mmol) in methanol (5 mL), 30% hydrogen peroxide (5.00 mmol) was added and the mixture was stirred at 50 °C for 1 h. Methanol was evaporated, the obtained residue was dissolved in DCM, followed by addition of manganese oxide and anhydrous magnesium sulfate. The mixture was dried for 24 h, filtered and evaporated. 

Method B: To a solution of diselenide (1.00 mmol) in methanol (5 mL), 30% hydrogen peroxide (5.00 mmol) was added and the mixture was stirred at 50 °C for 1 h. Methanol was evaporated, the obtained residue was dissolved in DCM, followed by addition of manganese oxide and anhydrous magnesium sulfate. Mixture was dried for 24 h, filtered and evaporated.

2-(N-ethylcarboxyamido)benzeneselenenic acid 10

Yield: 90%, 84%; mp 135–139 °C; ^1^H NMR (700 MHz, DMSO) δ = 1.25 (t, J = 7.7 Hz, 3H, CH_3_), 3.63–3.68 (m, 1H, N-CH_2_), 3.75–3.81 (m, 1H, N-CH_2_), 7.75 (dt, J_1_ = 0.7, J_2_ = 7.7 Hz, 1H, 1H_ar_), 7.82 (dt, J_1_ = 1.4, J_2_ = 7.7 Hz, 1H, 1H_ar_), 7.85 (d, J = 7.7 Hz, 1H, 1H_ar_), 8.16 (d, J = 7.7 Hz, 1H, 1H_ar_); ^13^C NMR (100.6 MHz, DMSO) δ = 15.83 (CH_3_), 37.00 (CH_2_), 127.25 (CH_ar_), 127.82 (CH_ar_), 131.36 (C_ar_), 132.70 (CH_ar_), 134.18 (CH_ar_), 147.64 (C_ar_), 168.31 (C=O); ^77^Se NMR (76 MHz, DMSO) δ = 1123.91 ppm; IR: 3242, 3074, 2973, 2928, 2872, 2363, 1703, 1686, 1619, 1587, 1567, 1549, 1445, 1380, 1360, 1313, 1291, 1271, 1246, 1145, 1115, 1090, 1056, 1024, 1007 cm^−1^; Elemental Anal. Calcd for C_9_H_11_NO_3_Se (260.90): C, 41.55; H, 4.26; N, 5.28 Found: C, 41.23; H, 4.18; N, 5.17.

2-(N-propylcarboxyamido)benzeneselenenic acid 11 

Yield: 65%, 84%; mp 138–142 °C; ^1^H NMR (700 MHz, DMSO) δ = 0.91 (t, J = 7.7 Hz, 3H, CH_3_), 1.63–1.71 (m, 2H, CH_2_), 3.56–3.61 (m, 1H, N-CH_2_), 3.66-3.70 (m, 1H, N-CH_2_), 7.60 (dt, J_1_ = 0.7, J_2_ = 7.0 Hz, 1H, 1H_ar_), 7.82 (dt, J_1_ = 1.4, J_2_ = 7.7 Hz, 1H, 1H_ar_), 7.85 (d, J = 7.7 Hz, 1H, 1H_ar_), 8.17 (d, J = 7.7 Hz, 1H, 1H_ar_); ^13^C NMR (100.6 MHz, DMSO) δ = 11.84 (CH_3_), 23.37 (CH_2_), 43.66 (CH_2_), 127.31 (CH_ar_), 127.82 (CH_ar_), 131.24 (C_ar_), 132.71 (CH_ar_), 134.20 (CH_ar_), 147.67 (C_ar_), 168.59 (C=O); ^77^Se NMR (76 MHz, DMSO) δ = 1126.91 ppm; IR: 3243, 3085, 2953, 2927, 2868, 1667, 1627, 1584, 1456, 1444, 1336, 1291, 1246, 1159, 1113, 1098, 1038, 1021 cm^−1^; Elemental Anal. Calcd for C_10_H_13_NO_3_Se (289.02): C, 45.84; H, 4.78; N, 5.11 Found: C, 43.53; H, 4.69; N, 5.27.

2-(N-butylcarboxyamido)benzeneselenenic acid 12

Yield: 60%, 47%; mp 121–125 °C; ^1^H NMR (700 MHz, DMSO) δ = 0.89 (t, J = 7.7 Hz, 3H, CH_3_), 1.34 (m, 2H, CH_2_), 1.58–1.67 (m, 2H, CH_2_), 3.58–3.63 (m, 1H, N-CH_2_), 3.70–3.74 (m, 1H, N-CH_2_), 7.42 (t, J = 7.0 Hz, 1H, 1H_ar_), 7.82 (t, J = 7.7 Hz, 1H, 1H_ar_), 7.84 (d, J = 7.7 Hz, 1H, 1H_ar_), 8.16 (d, J = 7.7 Hz, 1H, 1H_ar_); ^13^C NMR (100.6 MHz, DMSO) δ = 14.06 (CH_3_), 20.06 (CH_2_), 32.11 (CH_2_), 41.67 (CH_2_), 127.33 (CH_ar_), 127.78 (CH_ar_), 131.20 (C_ar_), 132.75 (CH_ar_), 134.23 (CH_ar_), 147.59 (C_ar_), 168.57 (C=O); ^77^Se NMR (76 MHz, DMSO) δ = 1126.33 ppm; IR: 3325, 3082, 2929,2870, 2851, 1668, 1586, 1586, 1446, 1444, 1357, 1324, 1299, 1248, 1231, 1182, 1150, 1112, 1065, 1022, 1007 cm^−1^; Elemental Anal. Calcd for C_11_H_15_NO_3_Se (289.02): C, 45.84; H, 5.25; N, 4.86 Found: C, 45.96; H, 5.34; N, 4.99.

2-(N-hexylcarboxyamido)benzeneselenenic acid 13

Yield: 33%, 36%; mp 84–87 °C; ^1^H NMR (700 MHz, DMSO) δ = 0.85 (s, 3H, CH_3_), 1.32–1.36 (m, 6H, 3 × CH_2_), 1.63–1.65 (m, 2H, CH_2_), 3.57–3.60 (m, 1H, N-CH_2_), 3.65–3.72 (m, 1H, N-CH_2_), 7.76 (t, J = 7.7 Hz, 1H, 1H_ar_), 7.81-7.85 (m, 2H, 2H_ar_), 8.16 (d, J = 7.7 Hz, 1H, 1H_ar_); ^13^C NMR (100.6 MHz, DMSO) δ = 14.36 (CH_3_), 22.45 (CH_2_), 26.48 (CH_2_), 29.97 (CH2), 31.35 (CH_2_), 41.93 (CH_2_), 127.33 (CH_ar_), 127.82 (CH_ar_), 131.21 (C_ar_), 132.76 (CH_ar_), 134.22 (CH_ar_), 147.82 (C_ar_), 168.58 (C=O); ^77^Se NMR (76 MHz, DMSO) δ = 1122.09 ppm; IR: 3243, 3076, 3058, 0958, 2926, 2856, 1682, 1622, 1585, 1568, 1552, 1460, 1445, 1376, 1329, 1298, 1250, 1215, 1108, 1039, 1026, 1010 cm^−1^; Elemental Anal. Calcd for C_13_H_19_NO_3_Se (317.05): C, 49.37; H, 6.06; N, 4.43 Found: C, 49.56; H, 6.14; N, 4.59.

2-(N-(3-methyl)butylcarboxyamido)benzeneselenenic acid 14

Yield: 52%, 72%; mp 119–123 °C; ^1^H NMR (700 MHz, DMSO) δ = 0.89–0.92 (m, 6H, 2 × CH_3_), 1.53–1.57 (m, 2H, CH_2_), 1.62–1.66 (m, 1H, CH), 3.61–3.65 (m, 1H, N-CH_2_), 3.72–3.77 (m, 1H, N-CH_2_), 7.76 (t, J = 7.0 Hz, 1H, 1H_ar_), 7.82 (dt, J_1_ = 0.7, J_2_ = 7.0 Hz, 1H, 1H_ar_), 7.85 (d, J = 7.0 Hz, 1H, 1H_ar_), 8.16 (d, J = 7.7 Hz, 1H, 1H_ar_); ^13^C NMR (100.6 MHz, DMSO) δ = 22.78 (CH_3_), 22.77 (CH_3_), 25.75 (CH), 38.92 (CH_2_), 40.30 (CH_2_), 127.32 (CH_ar_), 127.78 (CH_ar_), 131.21 (C_ar_), 132.75 (CH_ar_), 134.22 (CH_ar_), 147.57 (C_ar_), 168.52 (C=O); ^77^Se NMR (76 MHz, DMSO) δ = 1126.14 ppm; IR: 3317, 3060, 2956, 2895, 2868, 1666, 1585, 1459, 1445, 1385, 1366, 1328, 1301, 1266, 1248, 1170, 1146, 1128, 1113, 1047, 1032, 1011 cm^−1^; Elemental Anal. Calcd for C_12_H_17_NO_3_Se (303.04): C, 47.69; H, 5.67; N, 4.63 Found: C, 47.87; H, 5.58; N, 4.79.

2-(N-cyclohexylcarboxyamido)benzeneselenenic acid 15

Yield: 47%, 66%; mp 108–110 °C; ^1^H NMR (700 MHz, DMSO) δ = 1.15–1.22 (m, 1H), 1.28–1.40 (m, 2H, CH_2_), 1.56–1.70 (m, 3H), 1.75–1.83 (m, 2H, CH_2_), 2.01–2.05 (m, 2H, CH_2_), 4.07–4.12 (m, 1H, N-CH), 7.75 (dt, J_1_ = 0.7, J_2_ = 7.7 Hz, 1H, 1H_ar_), 7.82 (dt, J_1_ = 1.4, J_2_ = 7.7 Hz, 1H, 1H_ar_), 7.85 (dd, J_1_ = 1.4, J_2_ = 7.0 Hz, 1H, 1H_ar_), 8.13 (d, J = 7.7 Hz, 1H, 1H_ar_); ^13^C NMR (100.6 MHz, DMSO) δ = 25.44 (CH_2_), 25.79 (CH_2_), 25.91 (CH_2_), 33.71 (CH_2_), 33.91 (CH_2_), 54.38 (CH), 127.26 (CH_ar_), 127.55 (CH_ar_), 131.76 (C_ar_), 132.71 (CH_ar_), 134.16 (CH_ar_), 147.42 (C_ar_), 167.92 (C=O); ^77^Se NMR (76 MHz, DMSO) δ = 1113.88 ppm; IR: 3234, 2927, 2852, 1621, 1588, 1544, 1448, 1329, 1295, 1243, 1147, 1075 cm^−1^; Elemental Anal. Calcd for C_13_H_17_NO_3_Se (315.04): C, 49.69; H, 5.45; N, 4.46 Found: C, 49.40; H, 5.53; N, 4.32.

#### 2.2.2. Synthesis of 2-(N-alkylcarboxyamido)benzeneselenenic acid potassium salts 16–21

To a solution of benzeneselenic acid (1.00 mmol) in absolute ethanol (5 mL), potassium tert-butanolate (1.00 mmol) in absolute ethanol (2 mL) was added portionswise and stirred at room temperature for 1 h. The solution was evaporated and the residue was washed with diethyl ether (3 × 2 mL). All derivatives were obtained as yellow oil.

2-(N-ethylcarboxyamido)benzeneselenenic acid potassium salt 16

Yield: 98%; ^1^H NMR (700 MHz, D_2_O) δ = 1.12 (t, J = 7.7 Hz, 3H, CH_3_), 3.30–3.33 (m, 2H, N-CH_2_), 7.50 (dt, J_1_ = 1.4, J_2_ = 7.7 Hz, 1H, 1H_ar_), 7.61 (dd, J_1_ = 0.7, J_2_ = 7.7 Hz, 1H, 1H_ar_), 7.65 (dt, J_1_ = 1.4, J_2_ = 7.7 Hz, 1H, 1H_ar_), 7.99 (dd, J_1_ = 0.7, J_2_ = 7.7 Hz, 1H, 1H_ar_); ^13^C NMR (100.6 MHz, D_2_O) δ = 13.53 (CH_3_), 35.13 (CH_2_), 124.47 (CH_ar_), 127.36 (CH_ar_), 130.96 (CH_ar_), 132.34 (CH_ar_), 133.14 (C_ar_), 150.98 (C_ar_), 169.27 (C=O); ^77^Se NMR (76 MHz, D_2_O) δ = 1142.60 ppm; IR: 3192, 3052, 2971, 2932, 1628, 1587, 1543, 1444, 1376, 1359, 1314, 1146, 1051 cm^−1^; Elemental Anal. Calcd for C_9_H_10_KNO_3_Se (298.85): C, 36.25; H, 3.38; N, 4.70 Found: C, 36.58; H, 3.29; N, 4.85.

2-(N-propylcarboxyamido)benzeneselenenic acid potassium salt 17

Yield: 97%; ^1^H NMR (700 MHz, D_2_O) δ = 0.87 (t, J=7.0 Hz, 3H, CH_3_), 1.53–1.57 (m, 2H, CH_2_), 3.28 (t, J = 7.0 Hz, 2H, N-CH_2_), 7.52 (dt, J_1_ = 1.4, J_2_ = 7.7 Hz, 1H, 1H_ar_), 7.63 (dd, J_1_ = 1.4, J_2_ = 7.7 Hz, 1H, 1H_ar_), 7.67 (dt, J_1_ = 1.4, J_2_ = 7.7 Hz, 1H, 1H_ar_), 8.00 (dd, J_1_ = 1.4, J_2_ = 7.7 Hz, 1H, 1H_ar_); ^13^C NMR (100.6 MHz, D_2_O) δ = 10.73 (CH_3_), 21.89 (CH_2_), 41.83 (CH_2_), 124.47 (CH_ar_), 127.35 (CH_ar_), 130.94 (CH_ar_), 132.32 (CH_ar_), 133.23 (C_ar_), 150.94 (C_ar_), 169.53 (C=O); ^77^Se NMR (76 MHz, D_2_O) δ = 1142.45 ppm; IR: 3196, 3050, 2952, 2867, 1630, 1590, 1560, 1458, 1443, 1374, 1358, 1339, 1320, 1286, 1244, 1148 cm^−1^; Elemental Anal. Calcd for C_10_H_12_KNO_3_Se (312.96): C, 38.46; H, 3.87; N, 4.49 Found: C, 38.72; H, 3.93; N, 4.61.

2-(N-butylcarboxyamido)benzeneselenenic acid potassium salt 18

Yield: 96%; ^1^H NMR (700 MHz, D_2_O) δ = 0.83 (t, J=7.7 Hz, 3H, CH_3_), 1.28–1.33 (m, 2H, CH_2_), 1.49–1.55 (m, 2H, CH_2_), 3.31 (t, J = 7.7 Hz, 2H, N-CH_2_), 7.51 (t, J = 7.7 Hz, 1H, 1H_ar_), 7.61 (d, J = 7.7 Hz, 1H, 1H_ar_), 7.66 (dt, J_1_ = 0.7, J_2_ = 7.0 Hz, 1H, 1H_ar_), 8.00 (d, J = 7.7 Hz, 1H, 1H_ar_); ^13^C NMR (100.6 MHz, D_2_O) δ = 13.02 (CH_3_), 19.54 (CH_2_), 30.49 (CH_2_), 39.78 (CH_2_), 124.45 (CH_ar_), 127.33 (CH_ar_), 130.93 (CH_ar_), 132.29 (CH_ar_), 133.24 (C_ar_), 150.93 (C_ar_), 169.46 (C=O); ^77^Se NMR (76 MHz, D_2_O) δ = 1142.95 ppm; IR: 3216, 3061, 2957, 2930, 2870, 1628, 1588, 1547, 1460, 1440, 1318, 1258, 1148, 1125 cm^−1^; Elemental Anal. Calcd for C_10_H_12_KNO_3_Se (326.98): C, 40.49; H, 4.32; N, 4.29 Found: C, 40.23; H, 4.41; N, 4.44.

2-(N-hexylcarboxyamido)benzeneselenenic acid potassium salt 19

Yield: 89%; ^1^H NMR (700 MHz, D_2_O) δ = 0.76 (t, J = 7.0 Hz, 3H, CH_3_), 1.20–1.22 (m, 4H, 2 × CH_2_), 1.51–1.55 (m, 2H, CH_2_), 3.30 (t, J = 7.0 Hz, 2H, N-CH_2_), 7.50 (dt, J_1_ = 0.7, J_2_=7.7 Hz, 1H, 1H_ar_), 7.59 (dd, J_1_ = 1.4, J_2_ = 7.0 Hz, 1H, 1H_ar_), 7.65 (dt, J_1_ = 1.4, J_2_ = 7.0 Hz, 1H, 1H_ar_), 7.99 (d, J = 7.7 Hz, 1H, 1H_ar_); ^13^C NMR (100.6 MHz, D_2_O) δ=13.33 (CH_3_), 21.95 (CH_2_), 25.85 (CH_2_), 28.27 (CH_2_), 30.73 (CH_2_), 40.06 (CH_2_), 124.48 (CH_ar_), 127.30 (CH_ar_), 130.95 (CH_ar_), 132.31 (CH_ar_), 133.25 (C_ar_), 150.98 (C_ar_), 169.40 (C=O); ^77^Se NMR (76 MHz, D_2_O) δ = 1142.17 ppm; IR: 3218, 3058, 2955, 2926, 2856, 1626, 1588, 1543, 1459, 1436, 1400, 1376, 1314, 1252, 1120, 1012 cm^−1^; Elemental Anal. Calcd for C_13_H_18_KNO_3_Se (355.01): C, 44.06; H, 5.12; N, 3.95 Found: C, 44.38; H, 5.03; N, 4.19.

2-(N-(3-methyl)butylcarboxyamido)benzeneselenenic acid potassium salt 20

Yield: 91%; ^1^H NMR (700 MHz, D_2_O) δ = 0.85 (d, J = 7.0 Hz, 6H, 2 × CH_3_), 1.43–1.46 (m, 2H, CH_2_), 1.58–1.62 (m, 1H, CH), 3.34 (t, J = 7.0 Hz, 2H, N-CH_2_), 7.51 (dt, J_1_ = 0.7, J_2_ = 7.7 Hz, 1H, 1H_ar_), 7.60 (dd, J_1_ = 1.4, J_2_ = 7.7 Hz, 1H, 1H_ar_), 7.66 (dt, J_1_ = 1.4, J_2_ = 7.7 Hz, 1H, 1H_ar_), 8.00 (d, J = 7.0 Hz, 1H, 1H_ar_); ^13^C NMR (100.6 MHz, D_2_O) δ = 21.71 (2 × CH_3_), 25.28 (CH), 37.24 (CH_2_), 38.43 (CH_2_), 124.49 (CH_ar_), 127.34 (CH_ar_), 130.95 (CH_ar_), 132.32 (CH_ar_), 133.28 (C_ar_), 150.98 (C_ar_), 169.40 (C=O); ^77^Se NMR (76 MHz, D_2_O) δ = 1142.29 ppm; IR: 3189, 3057, 2954, 2929, 2869, 1627, 1588, 1545, 1461, 1365, 1315, 1260, 1227 cm^−1^; Elemental Anal. Calcd for C_12_H_16_KNO_3_Se (340.99): C, 42.35; H, 4.74; N, 4.12 Found: C, 42.13; H, 4.68; N, 4.27.

2-(N-cyclohexylcarboxyamido)benzeneselenenic acid potassium salt 21

Yield: 89%; ^1^H NMR (700 MHz, D_2_O) δ = 1.08 (m, 1H, CH), 1.22-1.31 (m, 4H, 2 × CH_2_), 1.53–1.54 (m, 1H, CH), 1.64–1.68 (m, 2H, CH_2_), 1.85–1.86 (m, 2H, CH_2_), 3.70–3.73 (m, 1H, CH), 7.49 (dt, J_1_ = 0.7, J_2_ = 7.7 Hz, 1H, 1H_ar_), 7.58 (dd, J_1_ = 0.7, J_2_=7.0 Hz, 1H, 1H_ar_), 7.65 (dt, J_1_ = 0.7, J_2_ = 7.7 Hz, 1H, 1H_ar_), 7.98 (d, J = 7.7 Hz, 1H, 1H_ar_); ^13^C NMR (176.08 MHz, D_2_O) δ = 24.16 (2 × CH_2_), 24.56 (CH_2_), 31.54 (2 × CH_2_), 49.40 (CH), 123.95 (CH_ar_), 126.97 (CH_ar_), 130.46 (CH_ar_), 131.79 (CH_ar_), 133.03 (C_ar_), 150.35 (C_ar_), 168.16 (C=O); ^77^Se NMR (76 MHz, D_2_O) δ = 1142.44 ppm; IR: 3183, 3057, 2927, 2853, 1628, 1587, 1540, 1449, 1369, 1336, 1300, 1257, 1072, 1053 cm^−1^; Elemental Anal. Calcd for C_13_H_16_KNO_3_Se (340.99): C, 44.32; H, 4.58; N, 3.98 Found: C, 44.55; H, 4.64; N, 3.82.

### 2.3. Antioxidant Activity Assay

Compounds 10–21 (0.1, 0,01 or 0,0075equiv.) and dithiothreitol DTT^red^ (0.15 mmol) were dissolved in 1.1 mL of CD_3_OD or D_2_O. Next, 30% H_2_O_2_ (0.15 mmol) was added and ^1^H NMR spectra were directly measured in specific time intervals. Following the changes in the integration on the ^1^H NMR spectra, the decay of the substrate was evaluated.

2.4. 3-(4,5-Dimethyldiazol-2-yl)-2,5 Diphenyl Tetrazolium Bromide (MTT) Viability Assay 

The MTT test was conducted by Mosmann methodology [21].

## 3. Results and Discussion

**Scheme 3:**
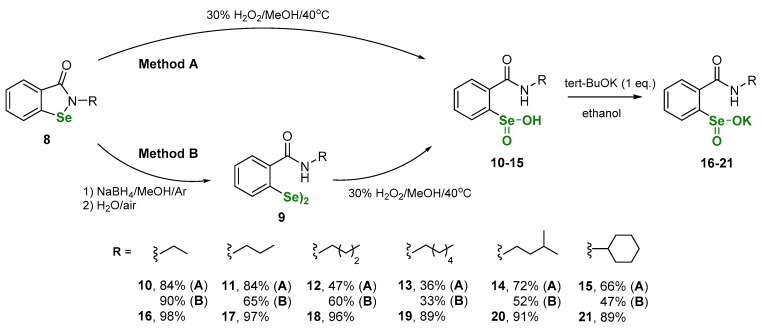
Sythesis of benzeneseleninic acids 10–15 and corresponding potassium salts 16–21.

The first step of this study involved the synthesis of N-alkyl benzeneselenenic acids with o-amido function. The compounds were obtained by two different methods based on the oxidation of N-alkylbenzisoselenazol-3(2H)-ones 8 - method A, or corresponding diselenides 9 - method B. Derivatives 8 and 9 were synthetized according to our previously published procedures [22,23,24,25]. The overall yields of both methods A and B were comparable. Next, benzeneseleninic acids 10-15 were transformed to the corresponding benzeneseleninic salts 16–21. The reaction was conducted using potassium tert-butanolate in anhydrous ethanol (Scheme 3).

The structures of both, benzeneseleninic acids and their potassium salts were fully characterized by ^1^H, ^13^C and ^77^Se NMR. The spectra were obtained in deuterated dimethyl sulfoxide (for compounds 10–15) and water (16–21). According to the best of our knowledge, NMR spectra of benzeneseleninic acids salts have never been published before.

**Figure 1:**
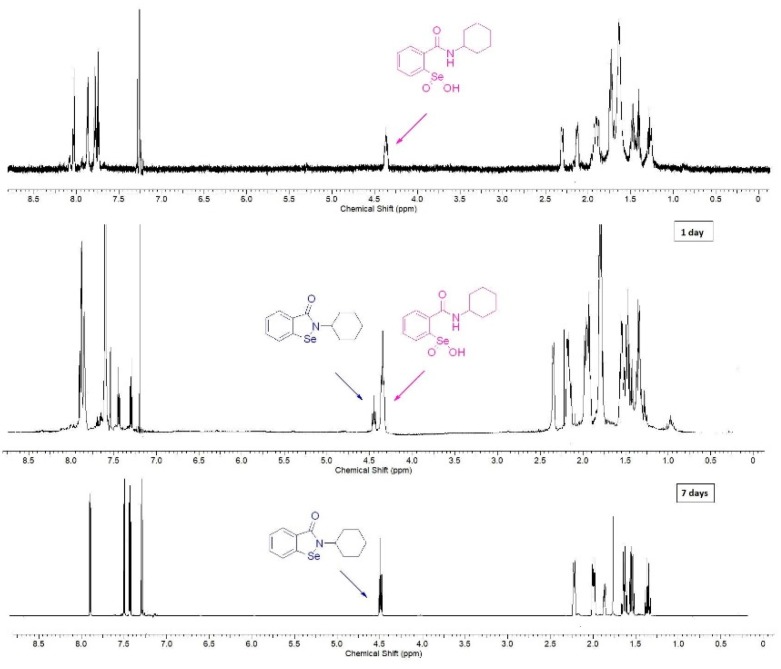
Decomposition of benzeneseleninic acid 15 to the corresponding benzisoselenazolone.

We observed that the stability of derivatives 10–15 in solution depends on the used solvent. Compound 15, dissolved in chloroform, was partially decomposed in 24 h and fully converted into the corresponding benzisoselenazolone in 7 days (Figure 1).

On the contrary, in DMSO, the decomposition was significantly slower and the sample could be stored for one week with no side-product formation. As it has been presented in Scheme 2, the transformation of benzeneseleninic acid 5 to ebselen 7 takes place through the formation of the selenenic acid 6. This could indicate that the rate of the reduction of R-SeOOH to R-SeOH can be influenced by the type of a solvent.

All derivatives were tested as antioxidants using the NMR activity assay proposed by Iwaoka and co-workers [26]. The efficiency of the peroxide scavenging potential was measured by the rate of dithiothreitol oxidation (DTT^red^ to DTT^ox^) in the presence of the selenocatalyst and equimolar amount of H_2_O_2_. Compounds 10–15 were applied in 0.1 molar equivalent and dissolved in deuterated methanol. Results are presented in Table 1.

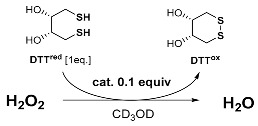



All benzeneseleninic acids 10–15 were more reactive than ebselen (N-phenylbenzisoselenazol-3(2H)-one) 7. The activity was higher for compounds 14 and 15, possessing more bulky substituents. The best result was obtained for N-cyclohexylbenzisoselenazol-3(2H)-one 15, for which only 2% of the substrate was observed after 60 minutes of reaction time. When the -SeOOH group was exchanged for -SeOOK, the activity increased drastically. The reaction was completed in 3 minutes. Consequently, all benzeneseleninic acid salts 16–21 were evaluated by the same procedure but using 0.01 equivalent of the Se-catalyst (Table 2).

In these conditions, almost no conversion was observed for ebselen. The N-ethyl derivative 16 was the most active one. Other results corresponded to the ones obtained for areneseleninic acids, with higher antioxidant potential observed for more sterically hindered compounds 20 and 21. The differences between antioxidant properties was initially evaluated by t-test. Due to various variance of tested groups, nonparametric Mann-Whitney test was additionally performed to confirm it. Both tests showed that the antioxidant properties of areneseleninic acid salts are significantly better than the well-known organoselenium antioxidant ebselen.

As salts 16–21 are soluble in water, they were additionally tested using the procedure proposed by Santi et. al. [27], in which deuterated water was used as a solvent. The reaction was very fast; thus, all compounds were used in only 0.0075 molar equivalents. The amount of a substrate was measured e for each catalyst after 2 min of reaction time (Table 3).

The best activity was observed for N-butyl 18 and N-ethyl derivative 16, as in the test performed in methanol (Table 2). The amount of DTT^red^ was 15% and 19% respectively, whereas when no catalyst was present still 93% of substrate remained in the reaction mixture. The rate of the process was also significantly improved when N-cyclohexylbenzeneseleninic acid salt 21 was applied. The test was also performed using benzeneseleninic acid potassium salt PhSeOOK. The obtained result indicates that the presence of an electron withdrawing group such as the o-amido function can improve the antioxidant properties.

Finally, the cytotoxic activity of acids 10–15 and salts 16–21 against breast cancer MCF-7 and human promyelocytic leukemia HL-60 cell lines was assessed using the cell viability assay (MTT) [21]. As a reference compound, we have used carboplatin. The results are shown in Table 4.

The highest cytotoxic activity against MCF-7 cells was found for N-cyclohexyl potassium salt 21 with IC_50_ 16.6 ± 1.1 µM. For most compounds, with the exception of acid 12, the conversion of -SeOOH to -SeOOK improved cytotoxicity. In HL-60 cell line the lowest IC_50_ value of 11.7 ± 1.0 µM was observed for N-ethyl benzeneseleninic acid 10. In general, with one exception (derivative 21), acids with -SeOOH group were more cytotoxic than the corresponding salts, in contrary to the IC_50_ values obtained for MCF-7 cell line.

## 4. Conclusions

This article presents the synthesis of the first water-soluble organoselenium antioxidants. A series of N-alkylcarboxyamidobenzeneseleninic acids and N-alkylcarboxyamidobenzeneseleninic acid potassium salts were obtained and evaluated as potential antioxidants and anticancer agents. All benzeneseleninic acid salts exhibited significant peroxide scavenging properties, both in methanol and water. The best obtained antioxidant, 2-(N-ethylcarboxyamido)benzeneselenenic acid potassium salt, used in only 0.01 equivalent, significantly increased the rate of H_2_O_2_ reduction, in comparison to the corresponding acid, and the well-known antioxidants, e.g., ebselen and the phenylseleninic acid potassium salt PhSeOOK. It indicates the necessity of adding the N-alkyl-o-amido function to the structure of the designed catalysts. The compounds can be applied in submicromolar amount what can decrease toxic side effects. The highest cytotoxic activity was observed for 2-(N-cyclohexyl-carboxyamido)benzeneselenenic acid potassium salt 21 in MCF-7 cells (IC_50_ 16.6 ± 1.1 µM) and for 2-(N-ethylcarboxyamido)benzeneselenenic acid 15 in HL-60 cells (IC_50_ 11.7 ± 1.0 µM). Generally, the compounds with -SeOOK moiety were more cytotoxic for breast cancer cells, whereas compounds with -SeOOH group were more potent against leukemia cells.

## Figures and Tables

**Table 1:** Results of the antioxidant activity measurement.

Catalyst (0.1 equiv.)	Remaining DTT^red^ (%)				
	3 min	5 min	15 min	30 min	60 min
Ebselen, 7	84	75	64	58	52
Benzseleninic acids					
10	89	86	78	67	51
11	81	75	70	63	51
12	85	82	75	65	47
13	89	85	69	46	19
14	76	56	38	24	12
15	85	64	37	18	2
Benzseleninic acid salts 16–21	0	0	0	0	0

**Table 2:** Results of the antioxidant activity measurement – 0.01 eq. of Se-catalyst.

Catalyst (0.01 equiv.)	Remaining DTT (%)					t-Test	Mann-Whitney Test
	3 min	5 min	15 min	30 min	60 min	p-value	p-value
Ebselen, 7	97	96	95	94	92	<0.001	<0.015
16	24	11	0	0	0	<0.005	<0.015
17	76	45	21	14	11	<0.005	<0.015
18	71	63	26	17	14	<0.01	<0.015
19	81	63	17	0	0	<0.001	<0.015
20	59	16	0	0	0	<0.005	<0.015
21	78	18	3	0	0	<0.001	<0.015

**Table 3:** Results of the antioxidant activity measurement performed in D_2_O – 0.0075 eq. of Se-catalyst.

Catalyst. (0.0075 equiv.)	16	17	18	19	20	21	PhSeOOK	No Catalyst
**DTT^red^ (%)**	19	47	15	46	79	26	49	93

**Table 4:** Cytotoxic activity of acids 1–14 and corresponding salts 16–20.

Structure	MCF-7 IC_50_ (µM)	HL-60 IC_50_ (µM)	Structure	MCF-7 IC_50_ (µM)	HL-60 IC_50_ (µM)
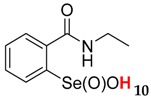	40.1 ± 1.2	11.7 ± 1.0	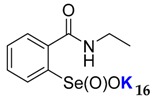	31.5 ± 1.2	56.8 ± 4.4
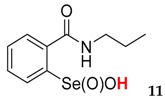	71.9 ± 4.1	23.5 ± 1.1	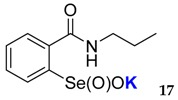	20.6 ± 2.5	63.1 ± 0.5
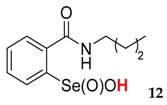	42.5 ± 0.8	62.1 ± 8.0	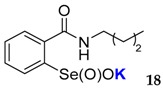	101.4 ± 2.3	92.6 ± 4.7
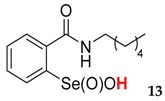	65.4 ± 0.9	113 ± 9.1	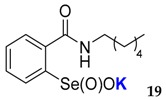	45 ± 0.2	200 ± 14.3
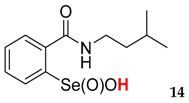	40.8 ± 4.5	51.5 ± 4.5	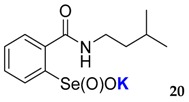	26.5 ± 3.5	72.5 ± 4.3
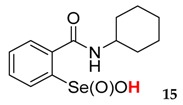	45.9 ± 5.3	70.8 ± 1.3	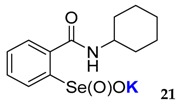	16.6 ± 1.1	42.1 ± 3.1
Carboplatin	3.80 ± 0.45	2.9 ± 0.1			

## Supplementary Materials

The following are available online at https://www.mdpi.com/1996-1944/13/3/661/s1, Figure S1. (a) ^1^H NMR, (b) ^13^C NMR, and (c) ^77^Se NMR spectra of 2-(N-ethylcarboxyamido)benzeneselenenic acid 10. Figure S2. (a) ^1^H NMR, (b) ^13^C NMR, and (c) ^77^Se NMR spectra of 2-(N-propylcarboxyamido)benzeneselenenic acid 11. Figure S3. (a) ^1^H NMR, (b) ^13^C NMR, and (c) ^77^Se NMR spectra of 2-(N-butylcarboxyamido)benzeneselenenic acid 12. Figure S4. (a) ^1^H NMR, (b) ^13^C NMR, and (c) ^77^Se NMR spectra of 2-(N-hexylcarboxyamido)benzeneselenenic acid 13. Figure S5. (a) ^1^H NMR, (b) ^13^C NMR, and (c) ^77^Se NMR spectra of 2-(N-(3-methyl)butylcarboxyamido)benzeneselenenic acid 14. Figure S6. (a) ^1^H NMR, (b) ^13^C NMR, and (c) ^77^Se NMR spectra of 2-(N-cyclohexylcarboxyamido)benzeneselenenic acid 15. Figure S7. (a) ^1^H NMR, (b) ^13^C NMR, and (c) ^77^Se NMR spectra of 2-(N-ethylcarboxyamido)benzeneselenenic acid potassium salt 16. Figure S8. (a) ^1^H NMR, (b) ^13^C NMR, and (c) ^77^Se NMR spectra of 2-(N-propylcarboxyamido)benzeneselenenic acid potassium salt 17. Figure S9. (a) ^1^H NMR, (b) ^13^C NMR, and (c) ^77^Se NMR spectra of 2-(N-butylcarboxyamido)benzeneselenenic acid potassium salt 18. Figure S10. (a) ^1^H NMR, (b) ^13^C NMR, and (c) ^77^Se NMR spectra of 22-(N-hexylcarboxyamido)benzeneselenenic acid potassium salt 19. Figure S11. (a) ^1^H NMR, (b) ^13^C NMR, and (c) ^77^Se NMR spectra of 2-(N-(3-methyl)butylcarboxyamido)benzeneselenenic acid potassium salt 20. Figure S12. (a) ^1^H NMR, (b) ^13^C NMR, and (c) ^77^Se NMR spectra of 2-(N-cyclohexylcarboxyamido)benzeneselenenic acid potassium salt 21.
