# Supplementary material for: Seleninic Acid Potassium Salts as Water-Soluble Biocatalysts with Enhanced Bioavailability
Source: Materials (Basel). 2020 Feb 2;13(3):661. doi: 10.3390/ma13030661 (PMC7040810; doi:10.3390/ma13030661)
Supplement: Supplementary file 1 [file materials-13-00661-s001.pdf]

materials

MDPI

Supporting Information

Seleninic Acid Potassium Salts as Water-Soluble Biocatalysts with Enhanced Bioavailability

Magdalena Obieziurska [1], Agata J. Pacuła [1], Anna Laskowska [1], Angelika Długosz-Pokorska [2], Anna Janecka [2] and Jacek Ścianowski [1,*,†]

1. NMR spectra

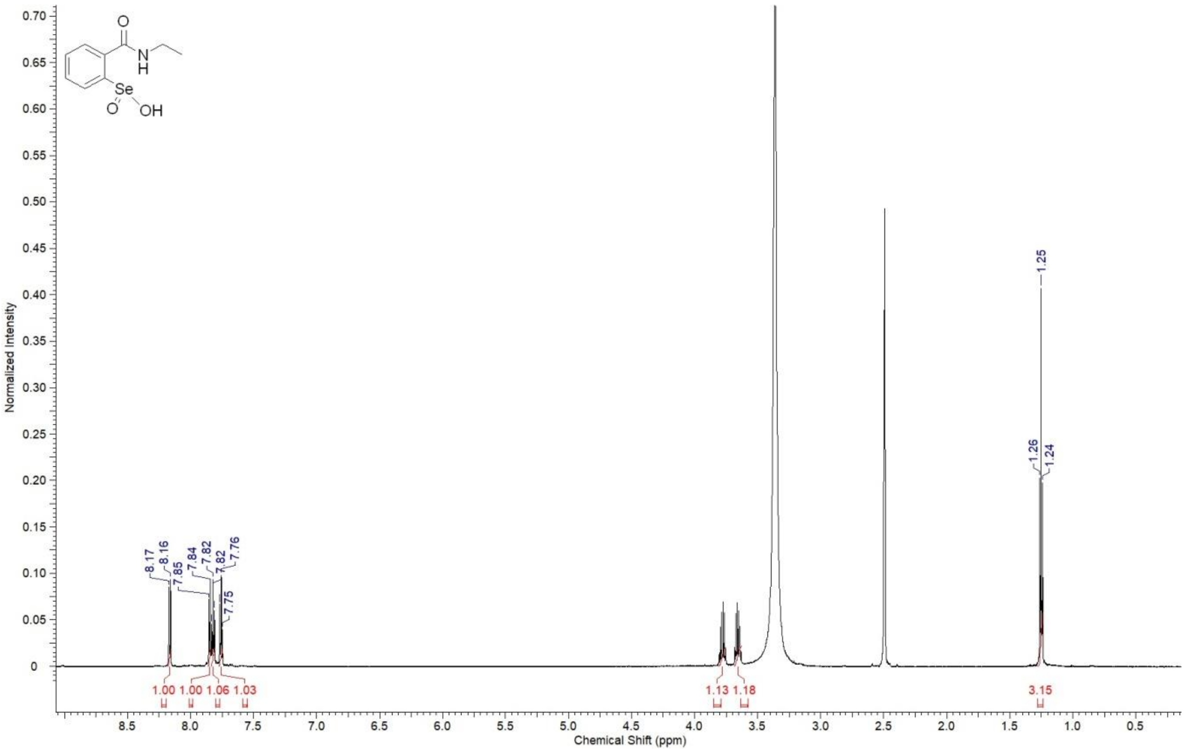

(a)

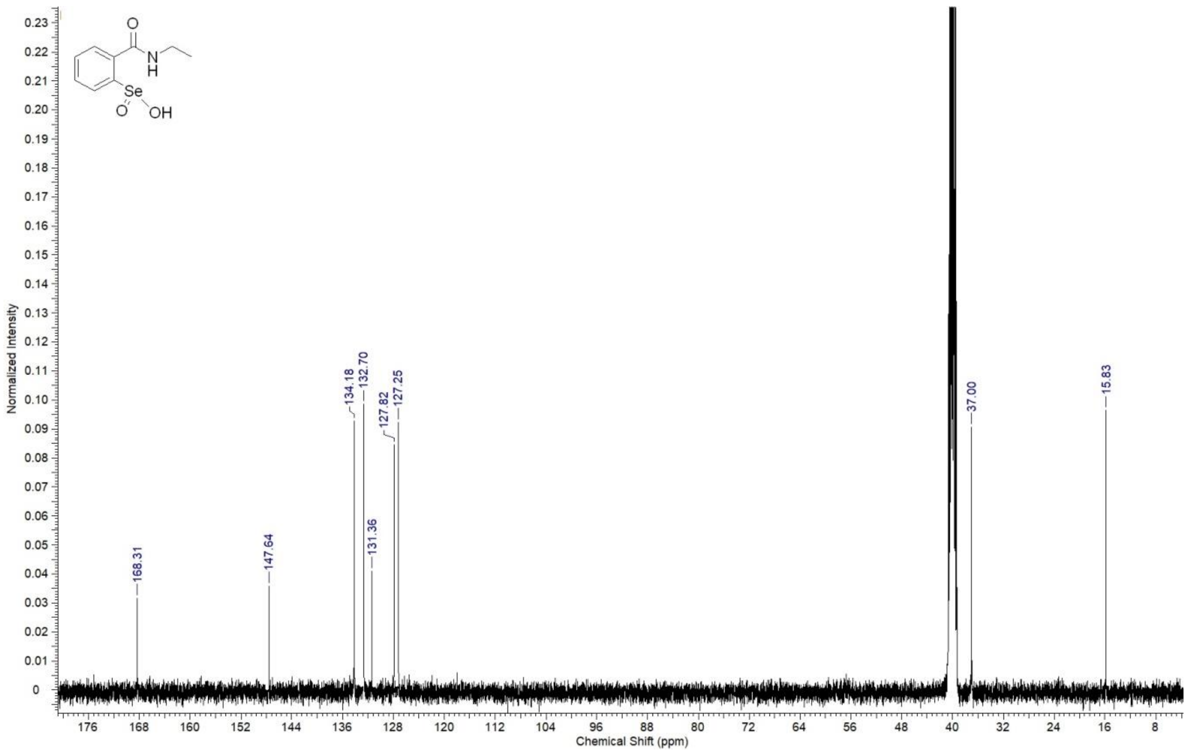

(b)

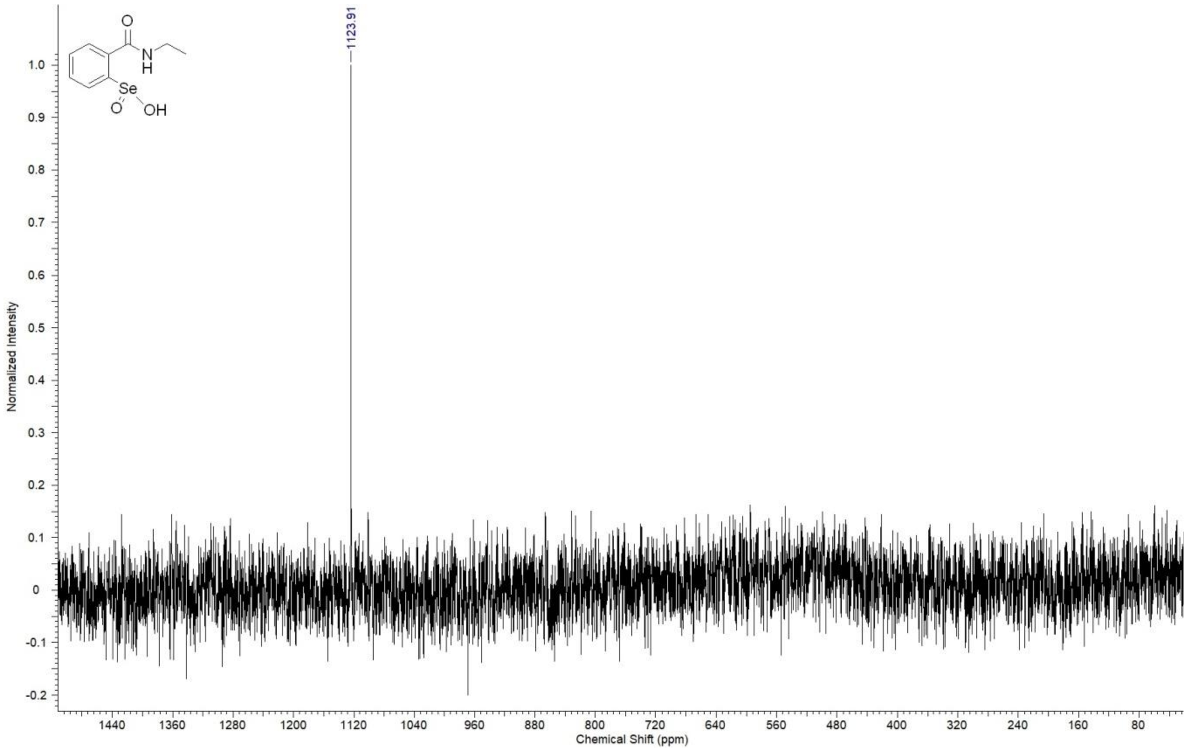

(c)

Figure S1. (**a**) ^{1}H NMR, (**b**) ^{13}C NMR, and (**c**) ^{77}Se NMR spectra of 2-(N-ethylcarboxyamido)-benzeneselenenic acid 10.

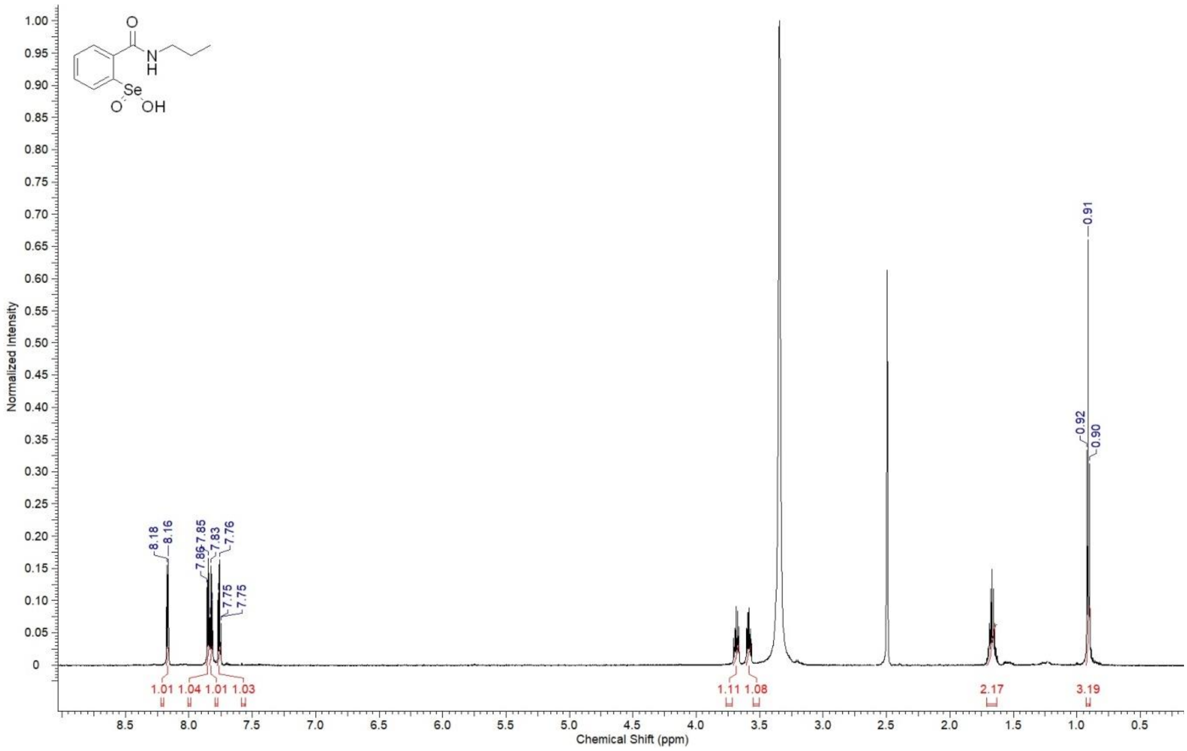

Normalized Intensity
Chemical Shift (ppm)
8.18
8.16
7.86
7.85
7.83
7.76
7.75
7.75
0.92
0.91
0.90
1.01
1.04
1.01
1.03
1.11
1.08
2.17
3.19
Se
OH

(a)

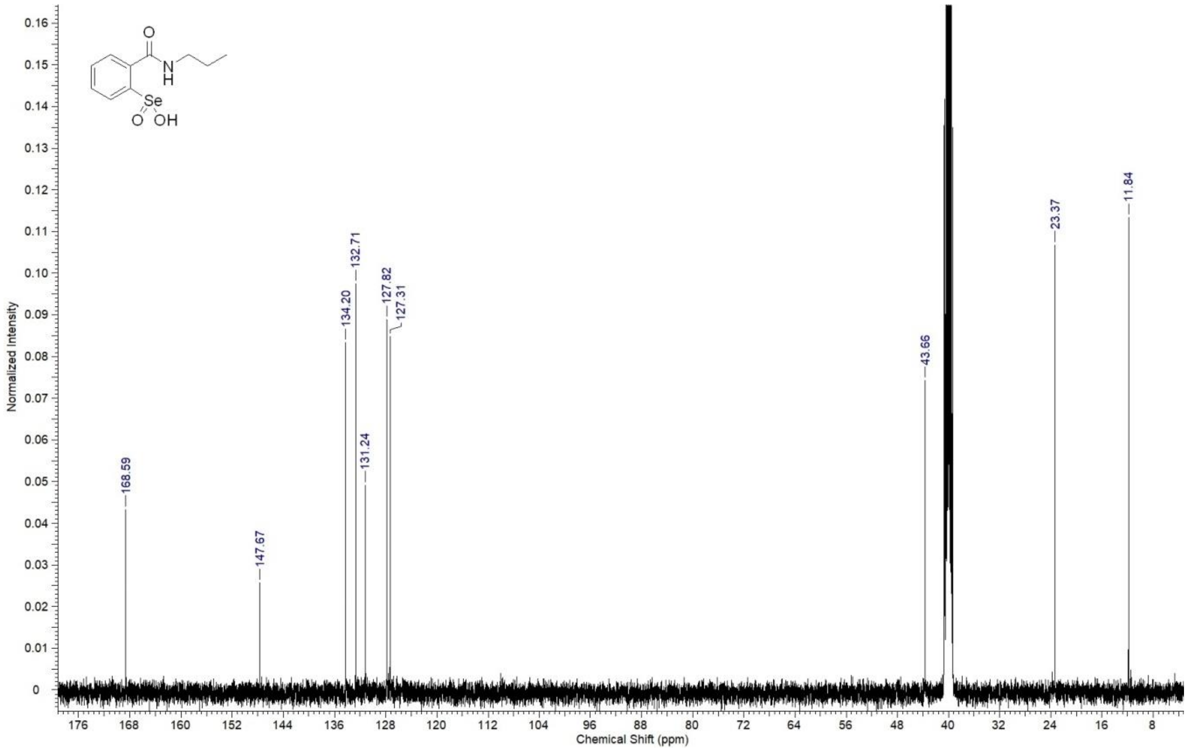

Normalized Intensity
Chemical Shift (ppm)
168.59
147.67
134.20
132.71
131.24
127.82
127.31
43.66
23.37
11.84
Se
OH

(b)

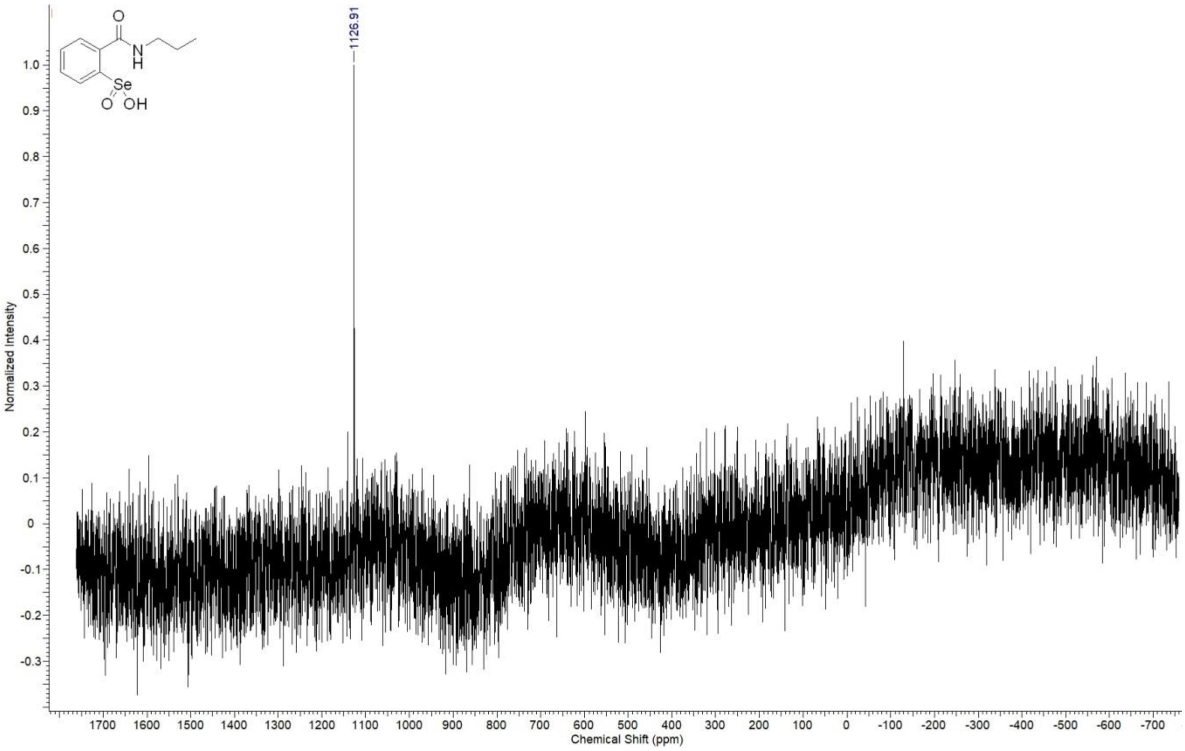

(c)

Figure S2. (**a**) ^{1}H NMR, (**b**) ^{13}C NMR, and (**c**) ^{77}Se NMR spectra of 2-(N-propylcarboxyamido)-benzeneselenenic acid 11.

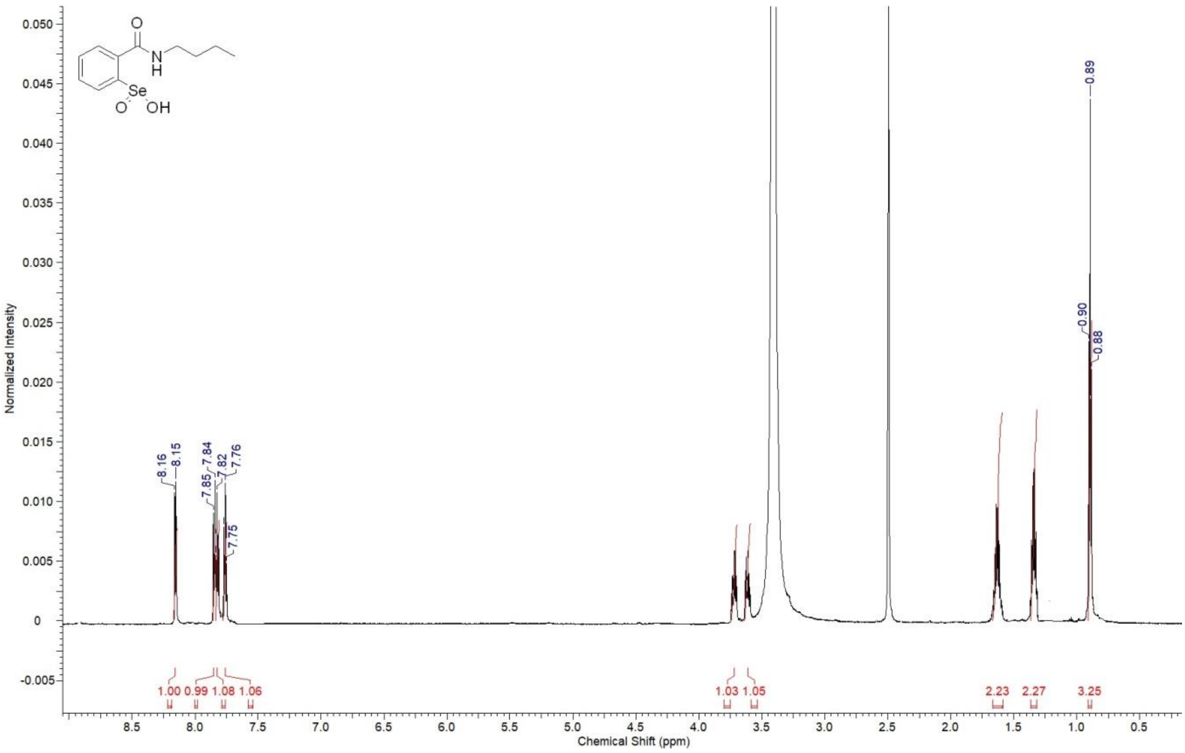

(a)

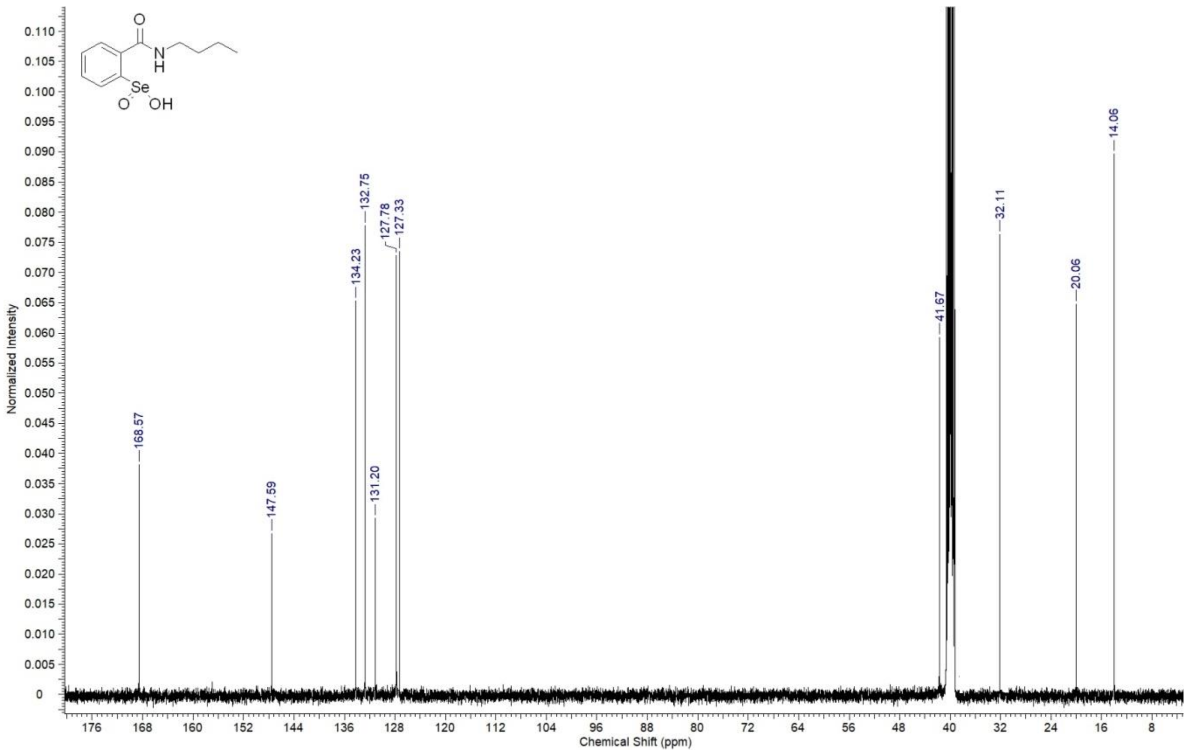

(b)

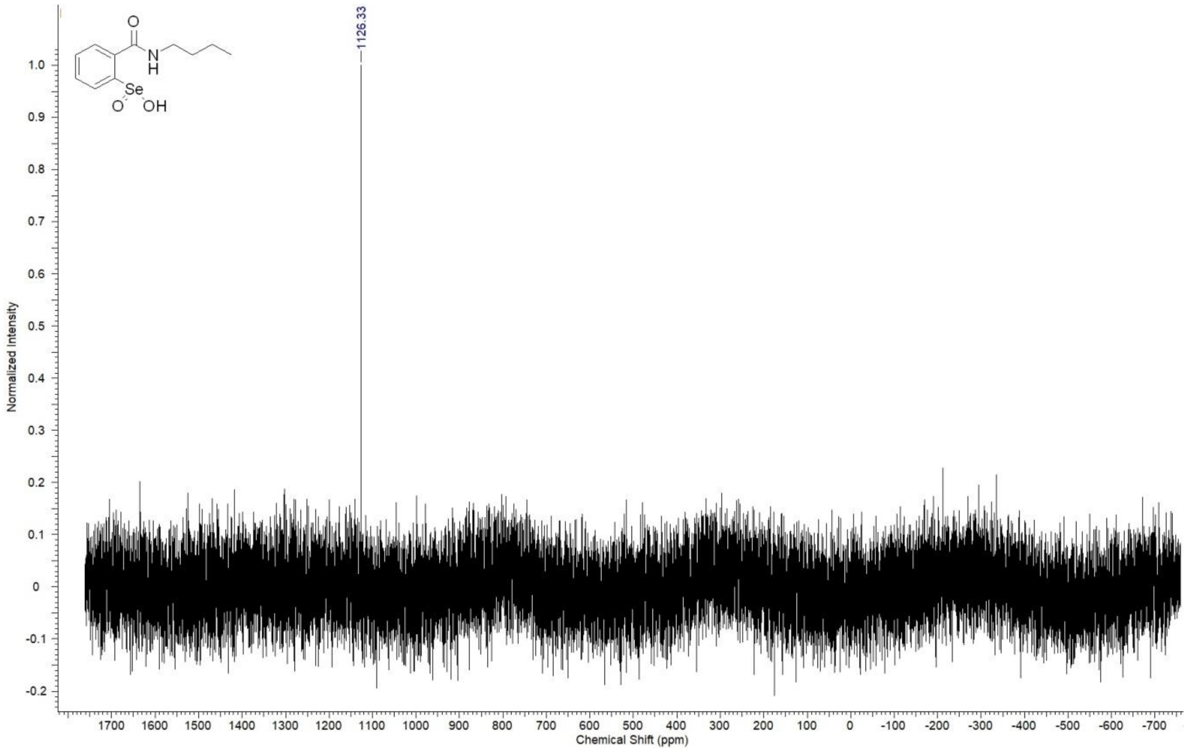

(c)

Figure S3. (**a**) ^{1}H NMR, (**b**) ^{13}C NMR, and (**c**) ^{77}Se NMR spectra of 2-(N-butylcarboxyamido)-benzeneselenenic acid 12.

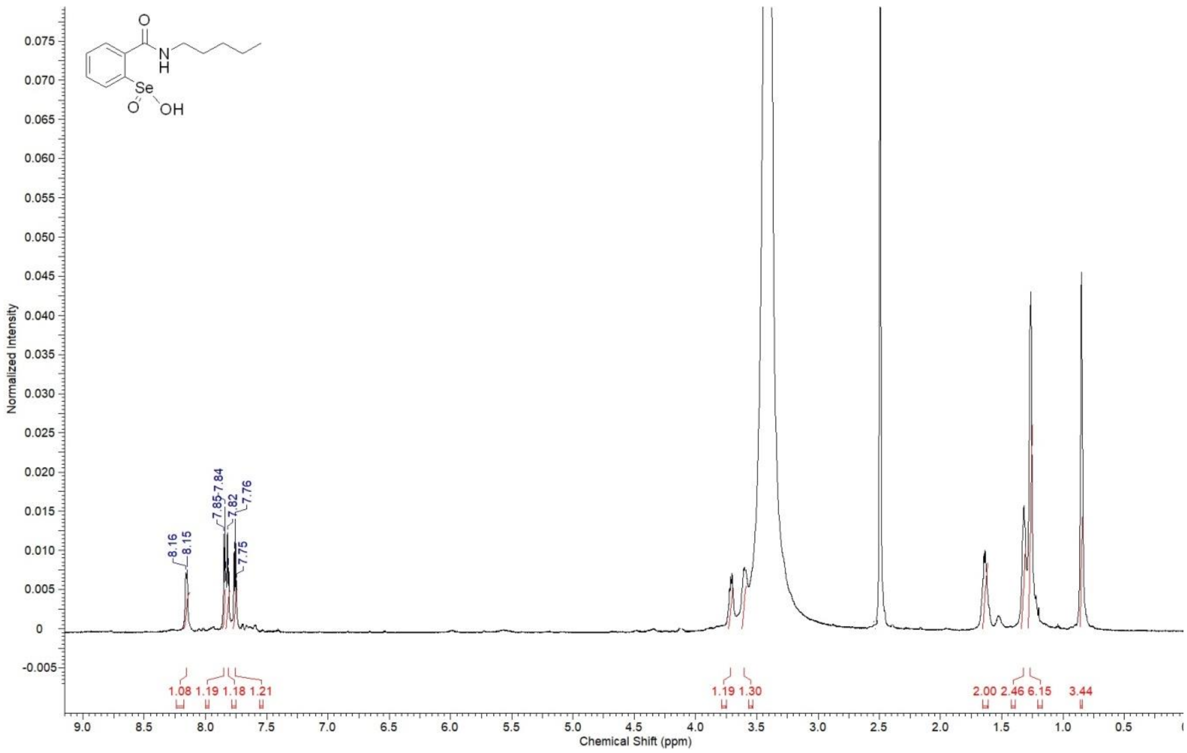
O
N
H
Se
O
OH
Normalized Intensity
8.16
8.15
7.85
7.84
7.82
7.76
7.75
1.08 1.19 1.18 1.21
1.19 1.30
2.00 2.46 6.15
3.44
Chemical Shift (ppm)

(a)

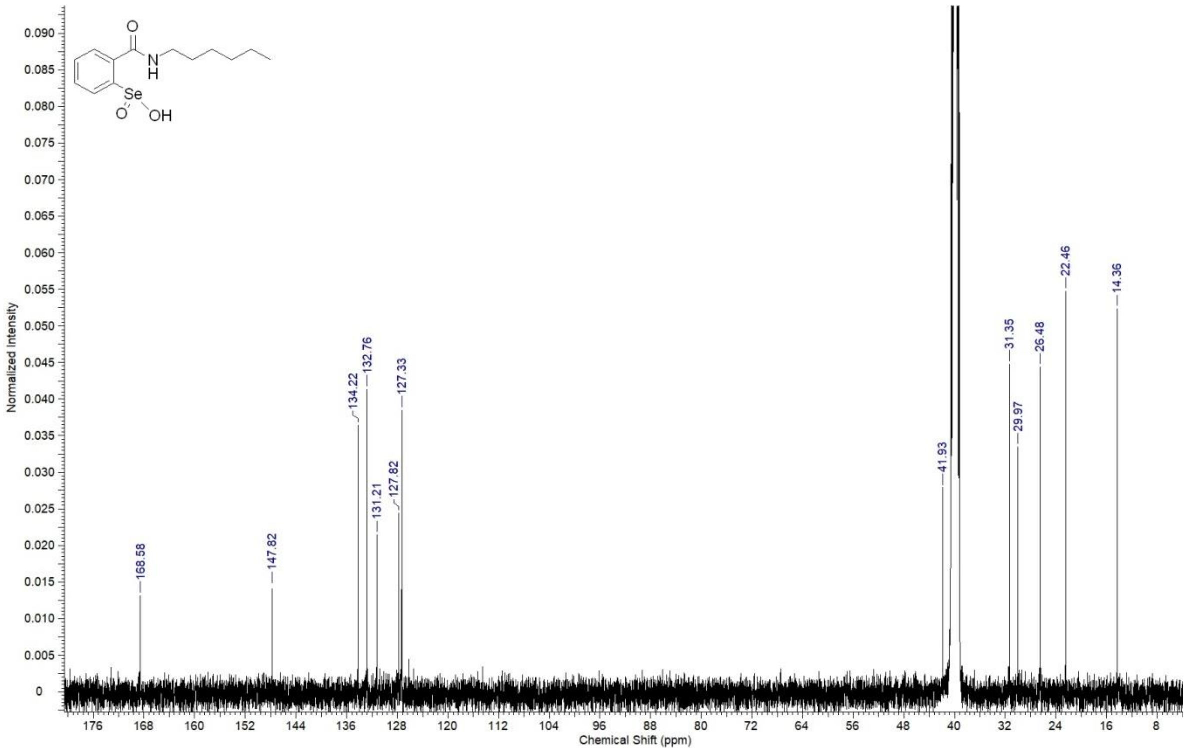
O
N
H
Se
O
OH
Normalized Intensity
168.58
147.82
134.22
132.76
131.21
127.82
127.33
41.93
31.35
29.97
26.48
22.46
14.36
Chemical Shift (ppm)

(b)

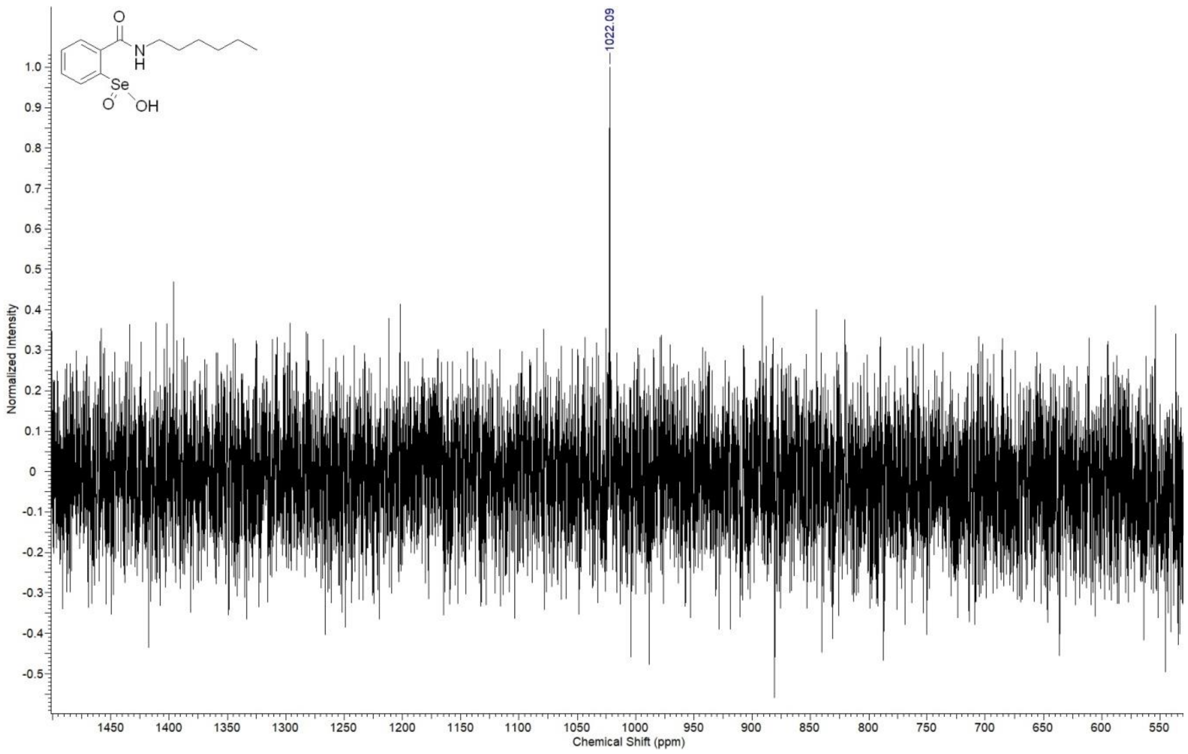

(c)

Figure S4. (**a**) ^{1}H NMR, (**b**) ^{13}C NMR, and (**c**) ^{77}Se NMR spectra of 2-(N-hexylcarboxyamido)-benzeneselenenic acid 13.

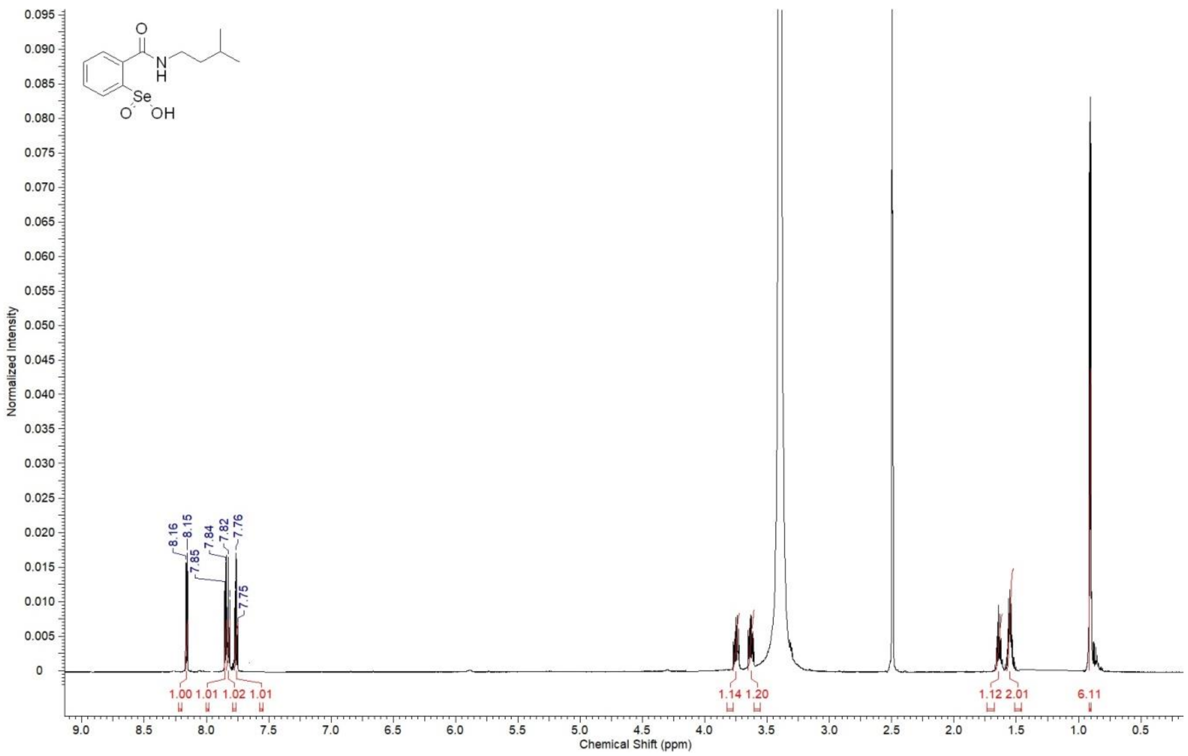

(a)

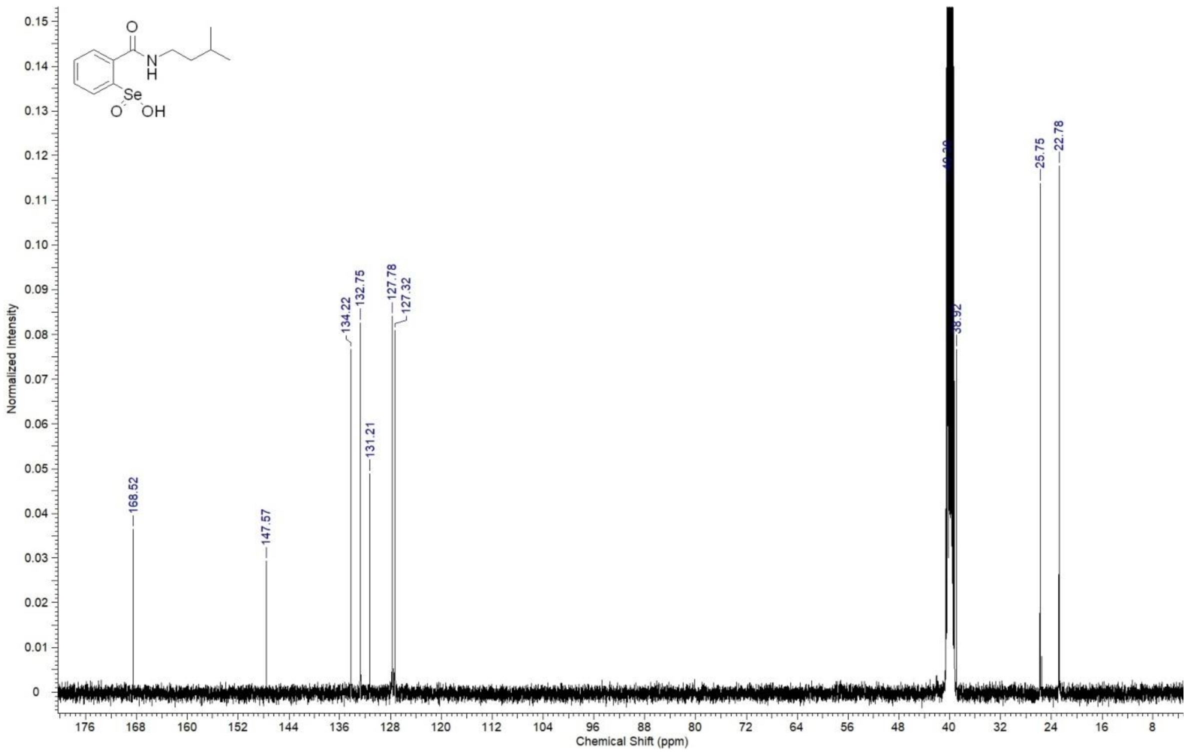

(b)

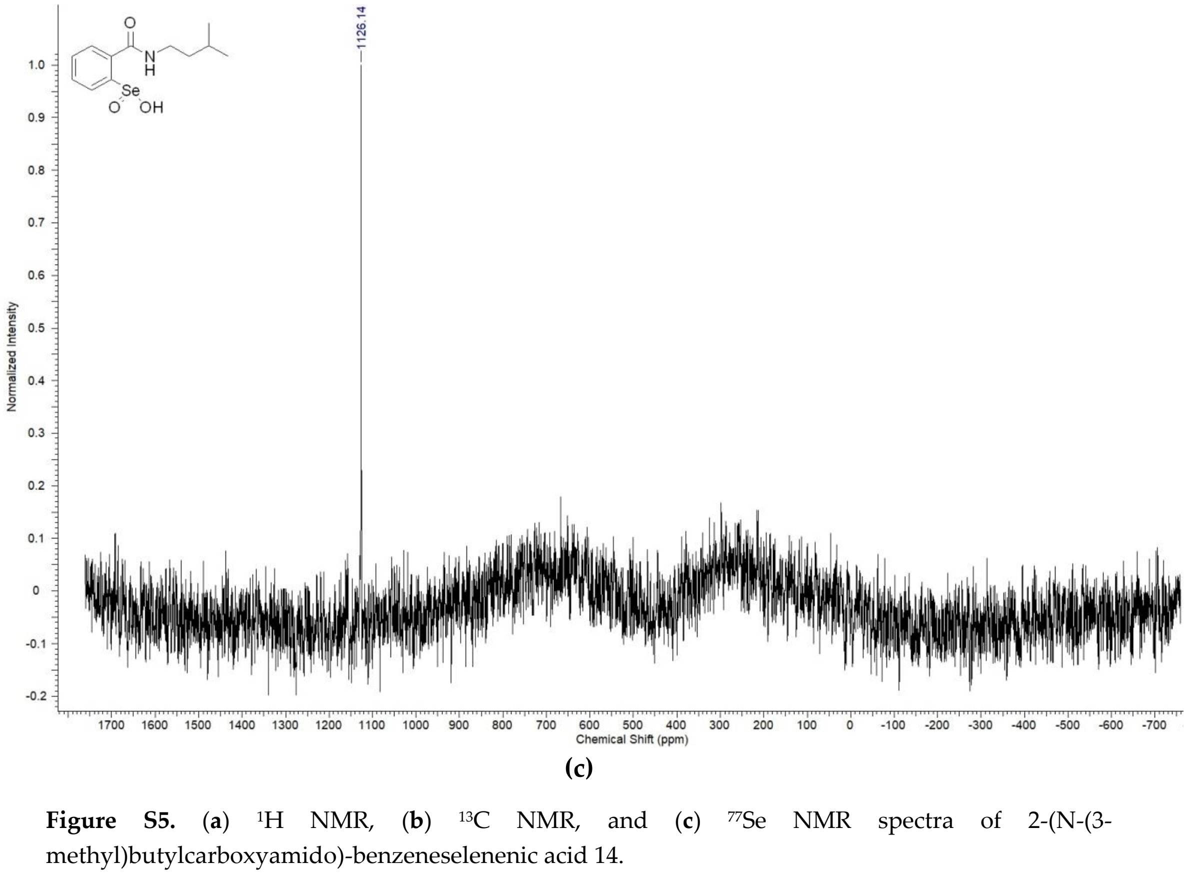

(c)

Figure S5. (**a**) ^{1}H NMR, (**b**) ^{13}C NMR, and (**c**) ^{77}Se NMR spectra of 2-(N-(3-methyl)butylcarboxyamido)-benzeneselenenic acid 14.

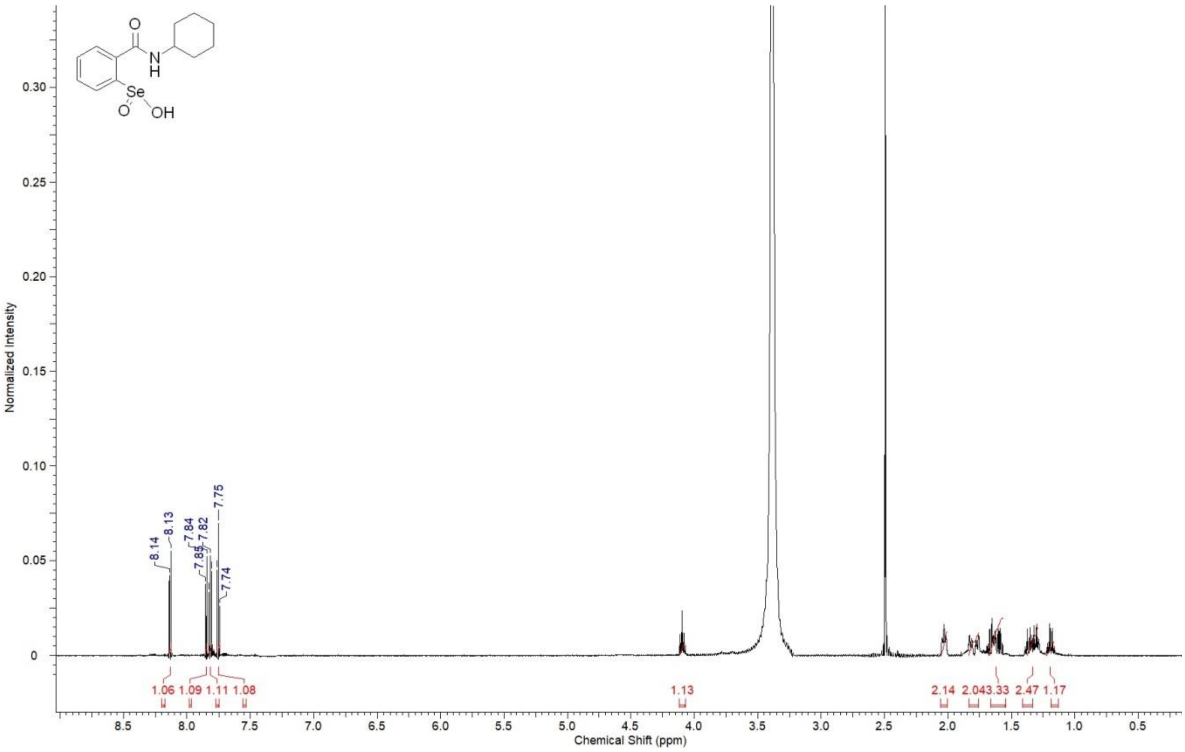
Se
OH
O
N
H
Normalized Intensity
8.14
8.13
7.84
7.83
7.82
7.75
7.74
1.06
1.09
1.11
1.08
1.13
2.14
2.04
3.33
2.47
1.17
Chemical Shift (ppm)

(a)

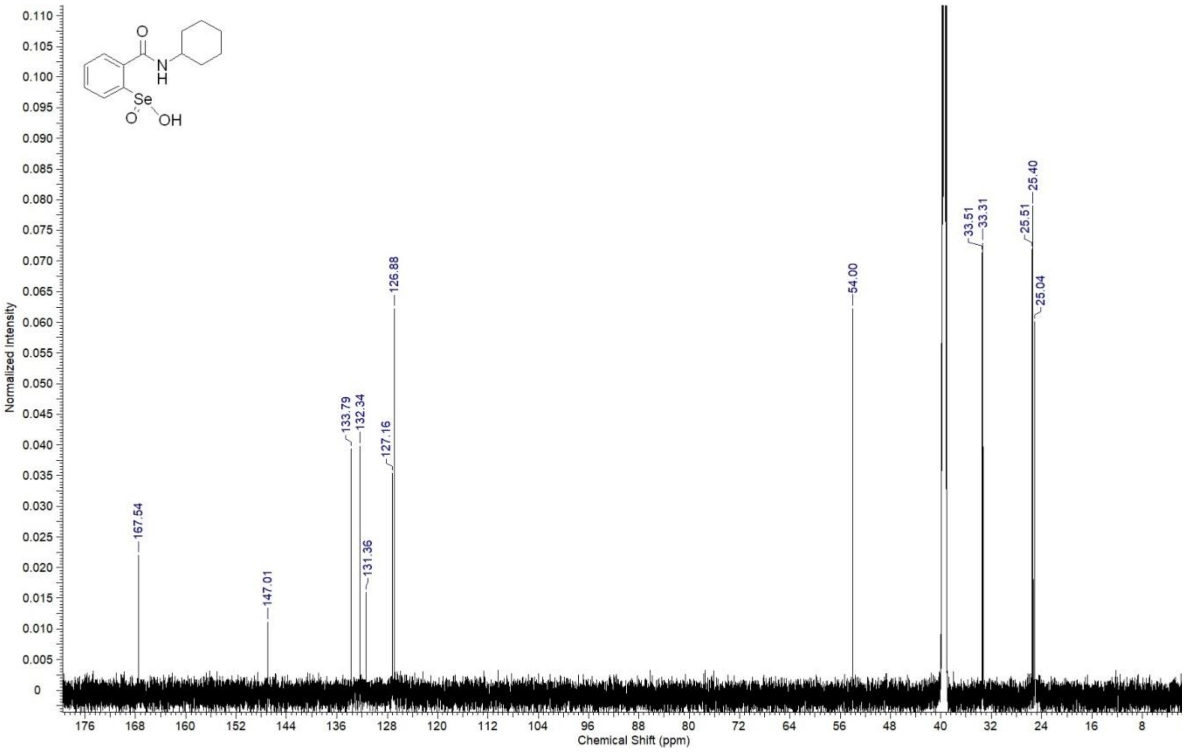
Se
OH
O
N
H
Normalized Intensity
167.54
147.01
133.79
132.34
131.36
127.16
126.88
54.00
33.51
33.31
25.51
25.40
25.04
Chemical Shift (ppm)

(b)

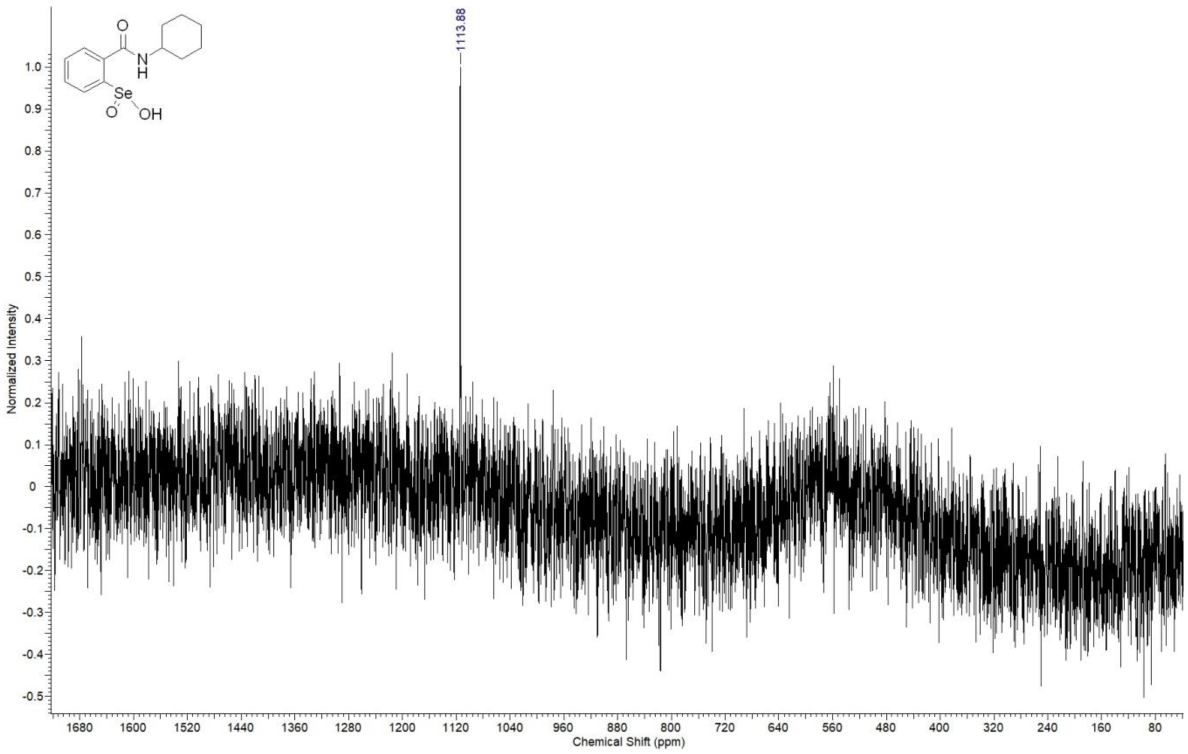

(c)

Figure S6. (**a**) ^{1}H NMR, (**b**) ^{13}C NMR, and (**c**) ^{77}Se NMR spectra of 2-(N-cyclohexylcarboxyamido)-benzeneselenenic acid 15.

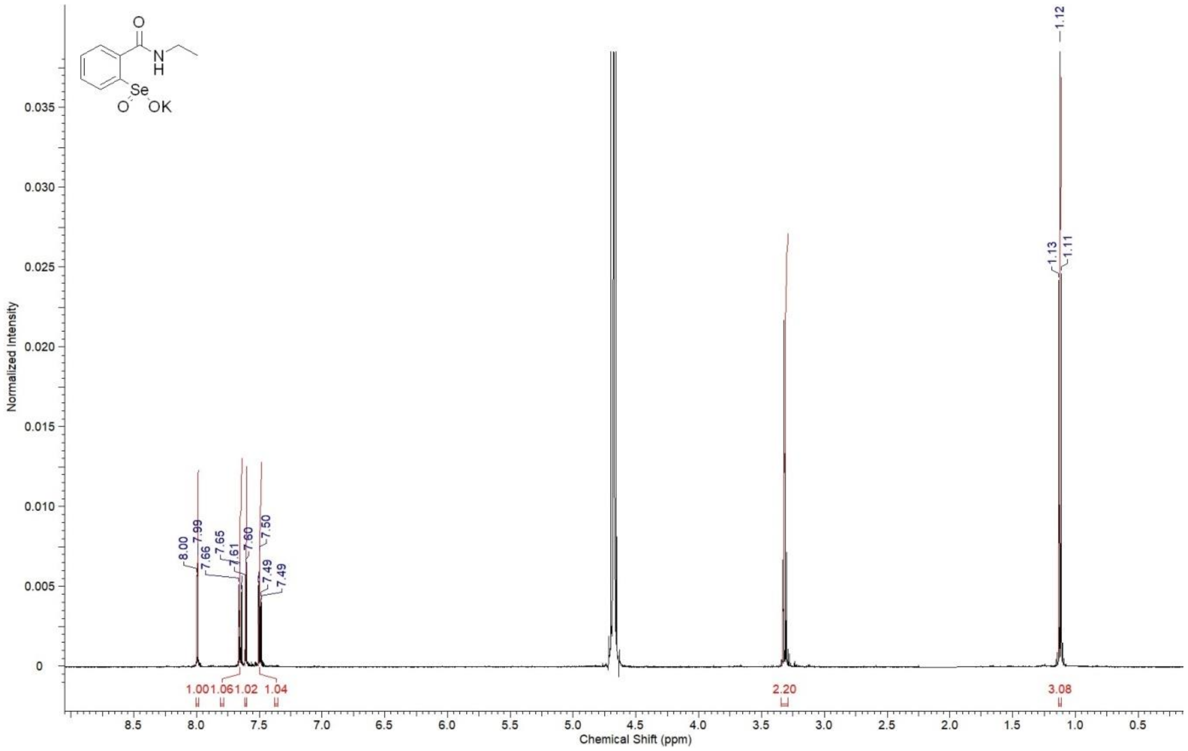

(a)

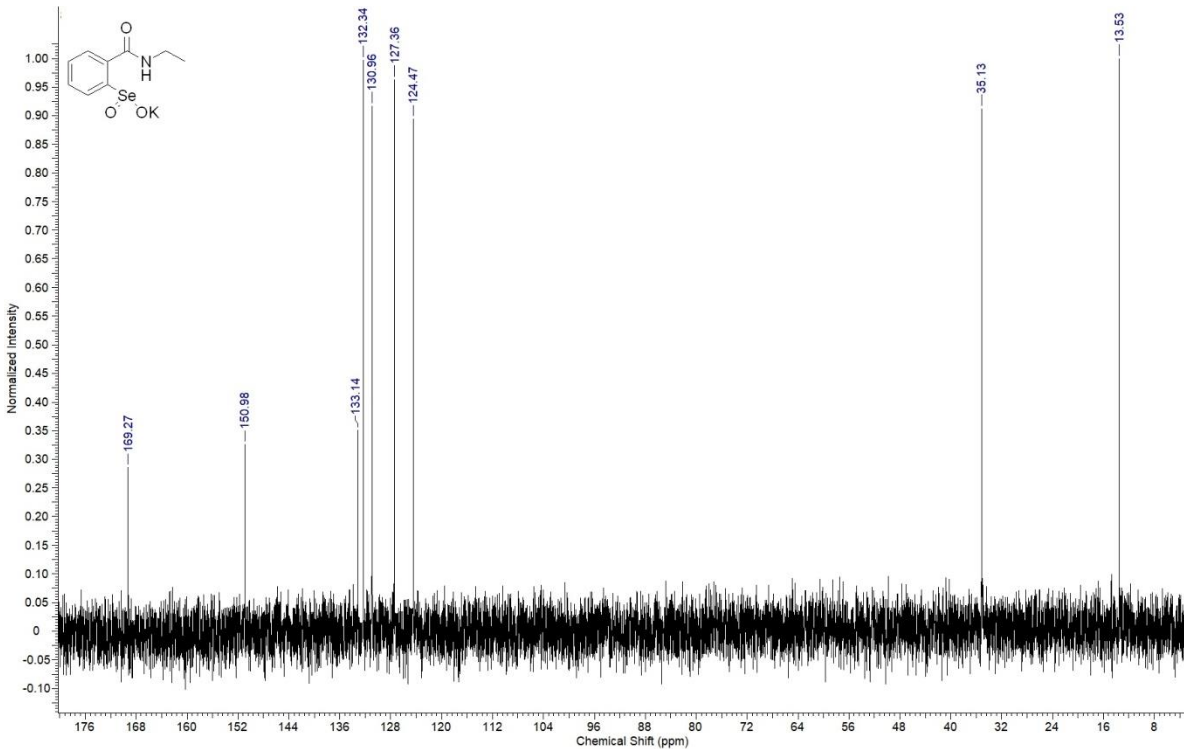

(b)

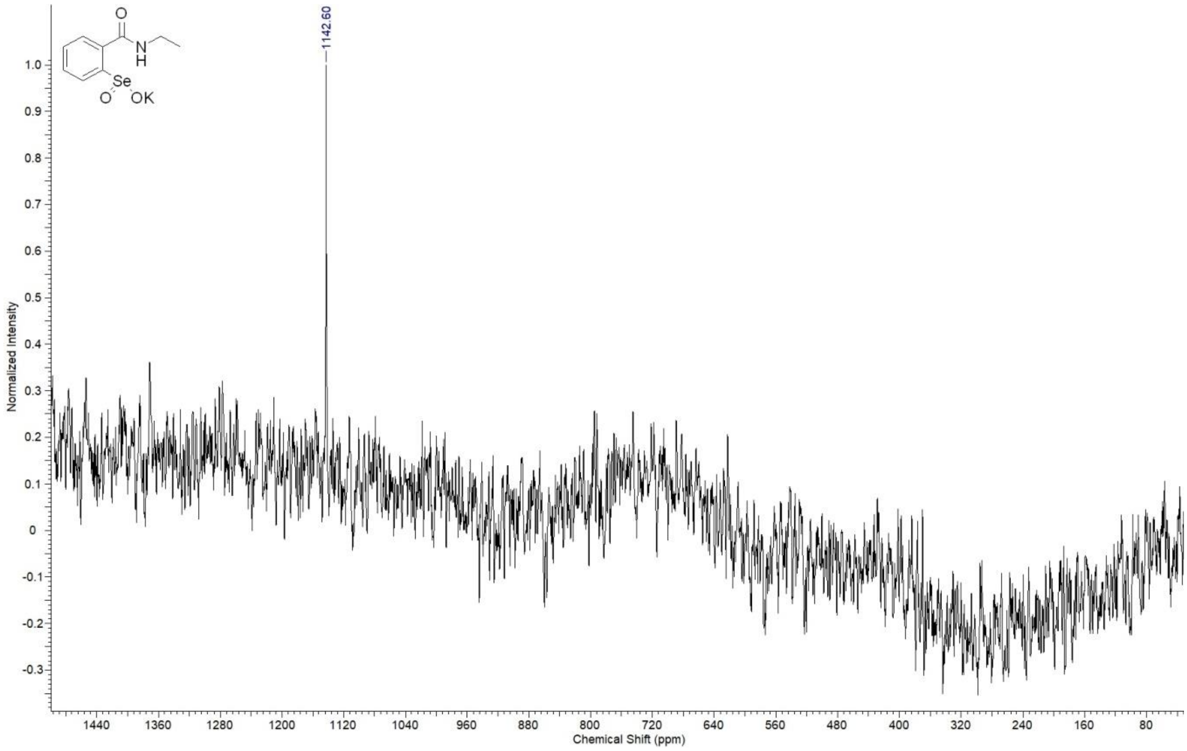

(c)

Figure S7. (**a**) ^{1}H NMR, (**b**) ^{13}C NMR, and (**c**) ^{77}Se NMR spectra of 2-(N-ethylcarboxyamido)-benzeneselenenic acid potassium salt 16.

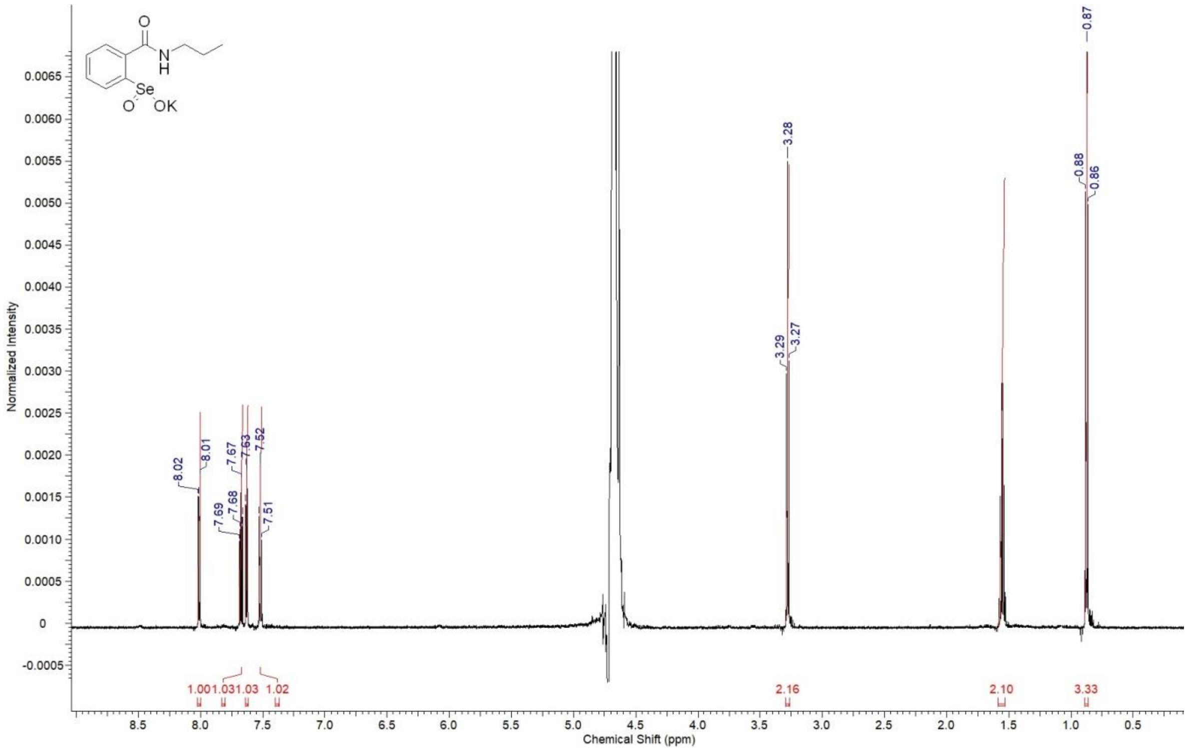
Se
OK
Normalized Intensity
Chemical Shift (ppm)
0.87
3.28
0.88
0.86
3.29
3.27
8.02
8.01
7.67
7.63
7.52
7.69
7.68
7.51
1.00
1.03
1.03
1.02
2.16
2.10
3.33

(a)

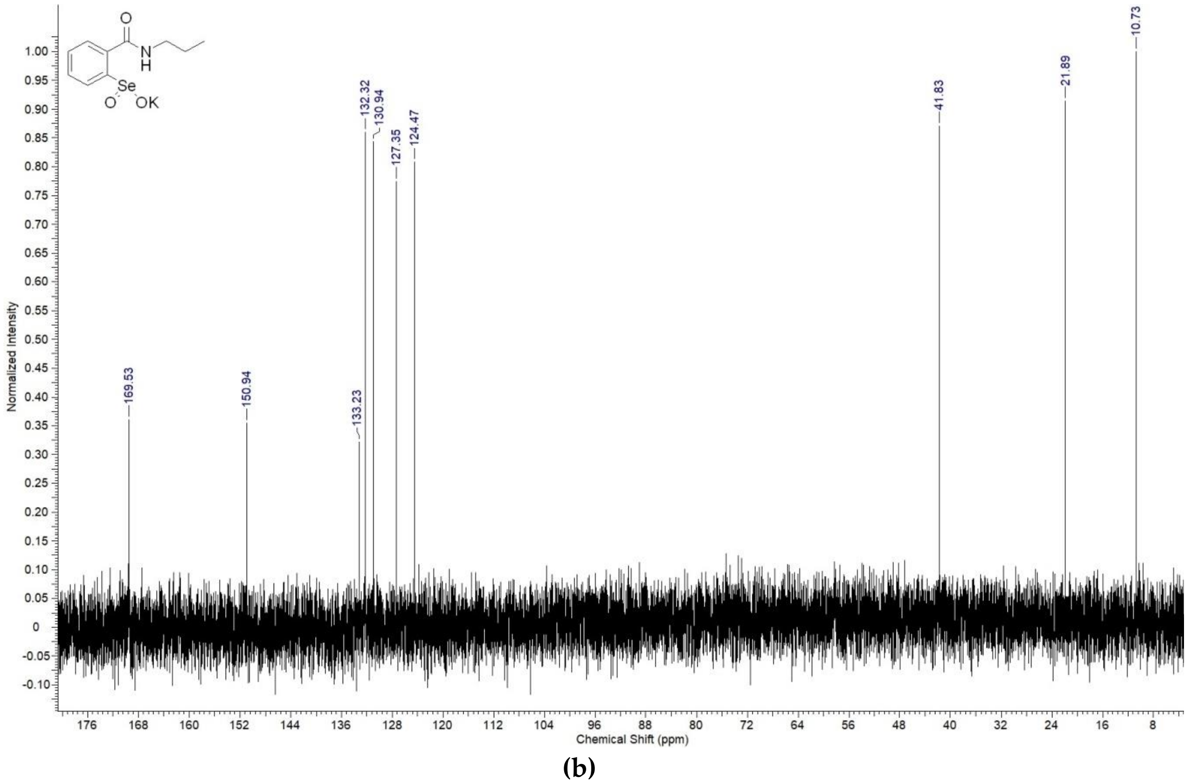
Se
OK
Normalized Intensity
Chemical Shift (ppm)
10.73
21.89
41.83
132.32
130.94
127.35
124.47
169.53
150.94
133.23

(b)

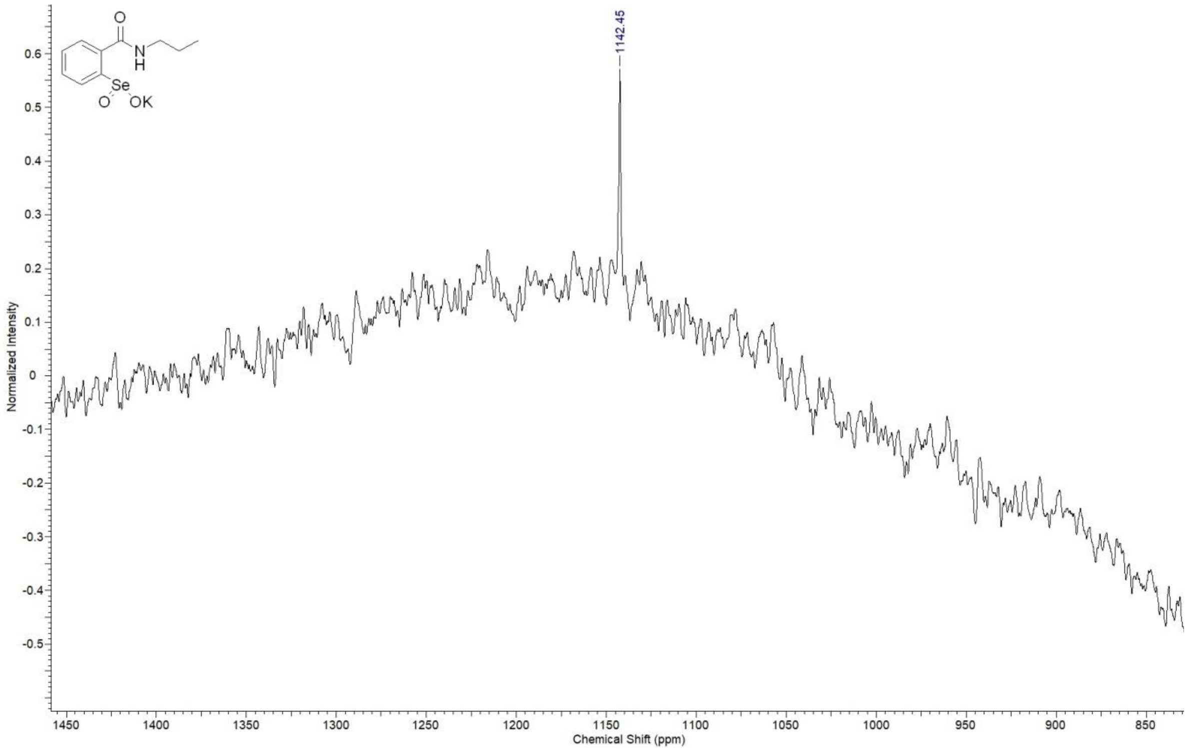

(c)

Figure S8. (**a**) ^{1}H NMR, (**b**) ^{13}C NMR, and (**c**) ^{77}Se NMR spectra of 2-(N-propylcarboxyamido)-benzeneselenenic acid potassium salt 17.

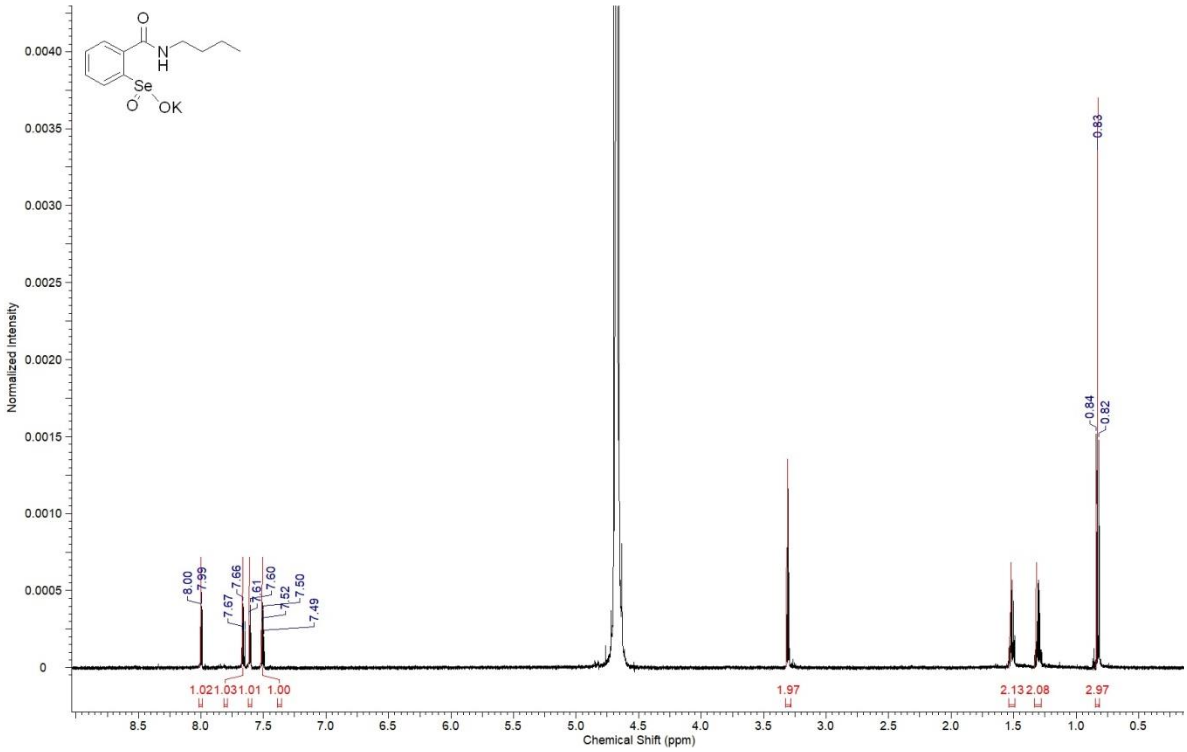

(a)

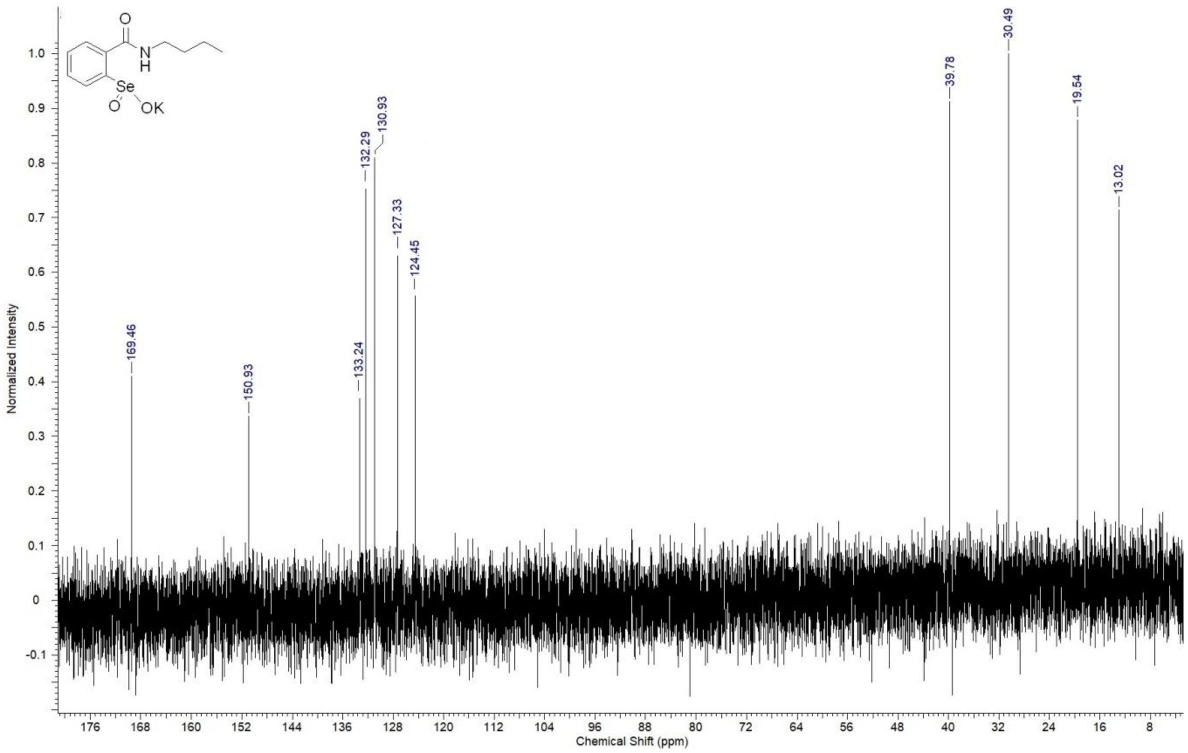

(b)

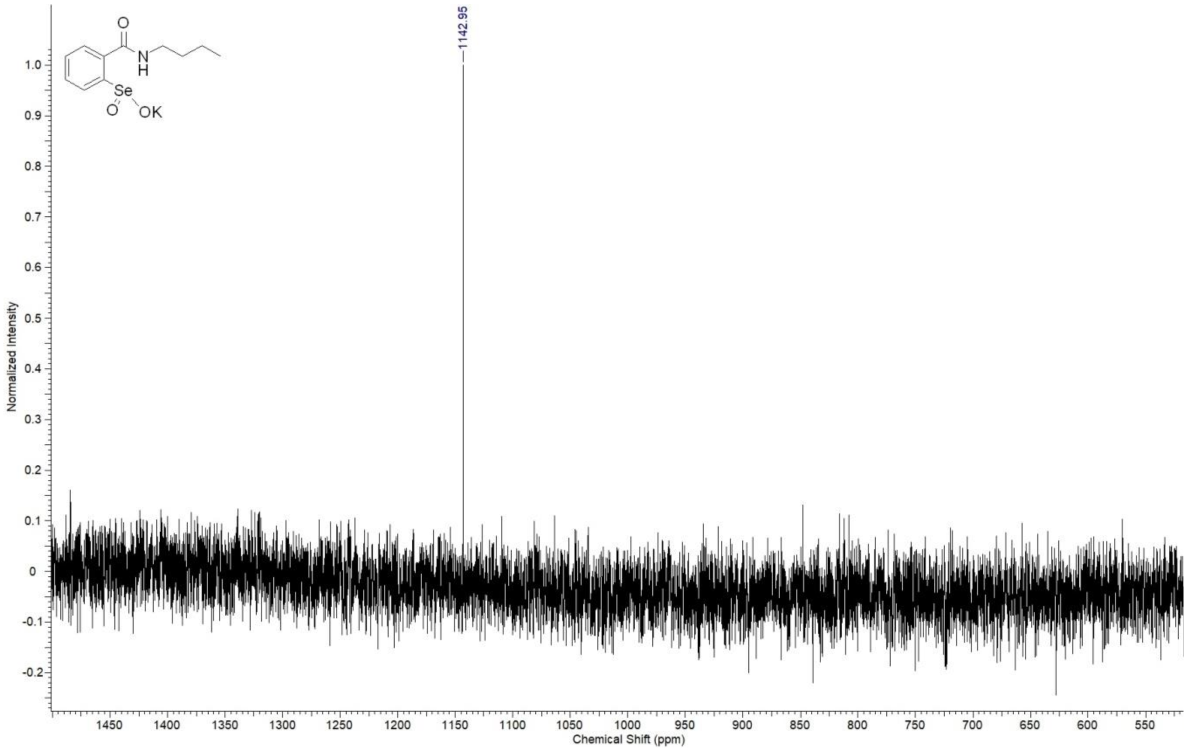

(c)

Figure S9. (**a**) ^{1}H NMR, (**b**) ^{13}C NMR, and (**c**) ^{77}Se NMR spectra of 2-(N-butylcarboxyamido)-benzeneselenenic acid potassium salt 18.

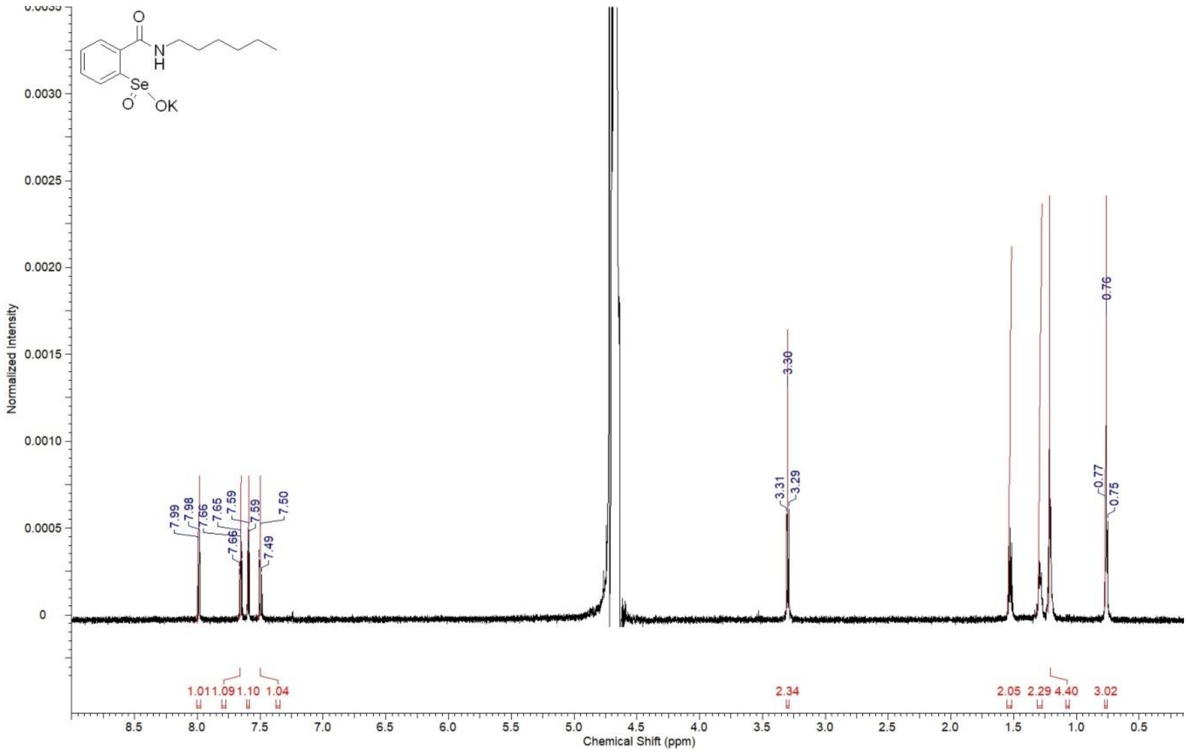
O
N
H
Se
O
OK
Normalized Intensity
0.0030
0.0025
0.0020
0.0015
0.0010
0.0005
0
7.99
7.98
7.66
7.65
7.59
7.66
7.59
7.50
7.49
3.30
3.31
3.29
0.76
0.77
0.75
1.01
1.09
1.10
1.04
2.34
2.05
2.29
4.40
3.02
8.5 8.0 7.5 7.0 6.5 6.0 5.5 5.0 4.5 4.0 3.5 3.0 2.5 2.0 1.5 1.0 0.5
Chemical Shift (ppm)

(a)

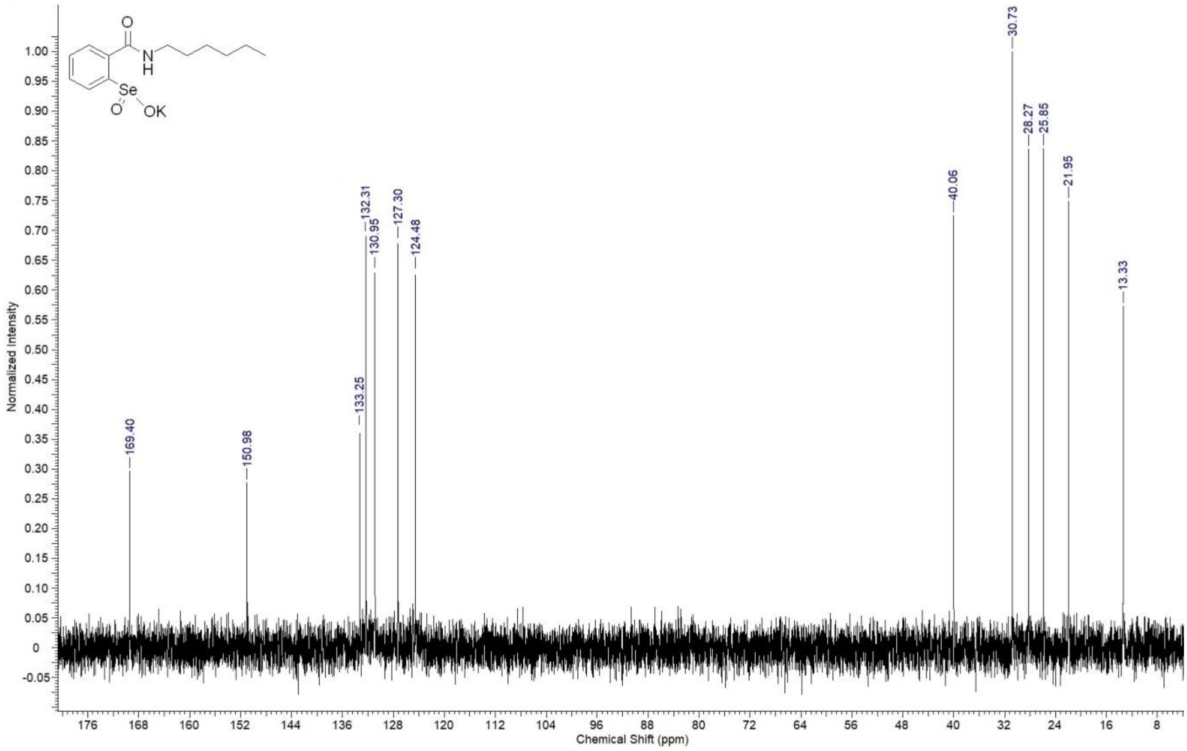
O
N
H
Se
O
OK
Normalized Intensity
1.00
0.95
0.90
0.85
0.80
0.75
0.70
0.65
0.60
0.55
0.50
0.45
0.40
0.35
0.30
0.25
0.20
0.15
0.10
0.05
0
-0.05
169.40
150.98
133.25
132.31
130.95
127.30
124.48
40.06
30.73
28.27
25.85
21.95
13.33
176 168 160 152 144 136 128 120 112 104 96 88 80 72 64 56 48 40 32 24 16 8
Chemical Shift (ppm)

(b)

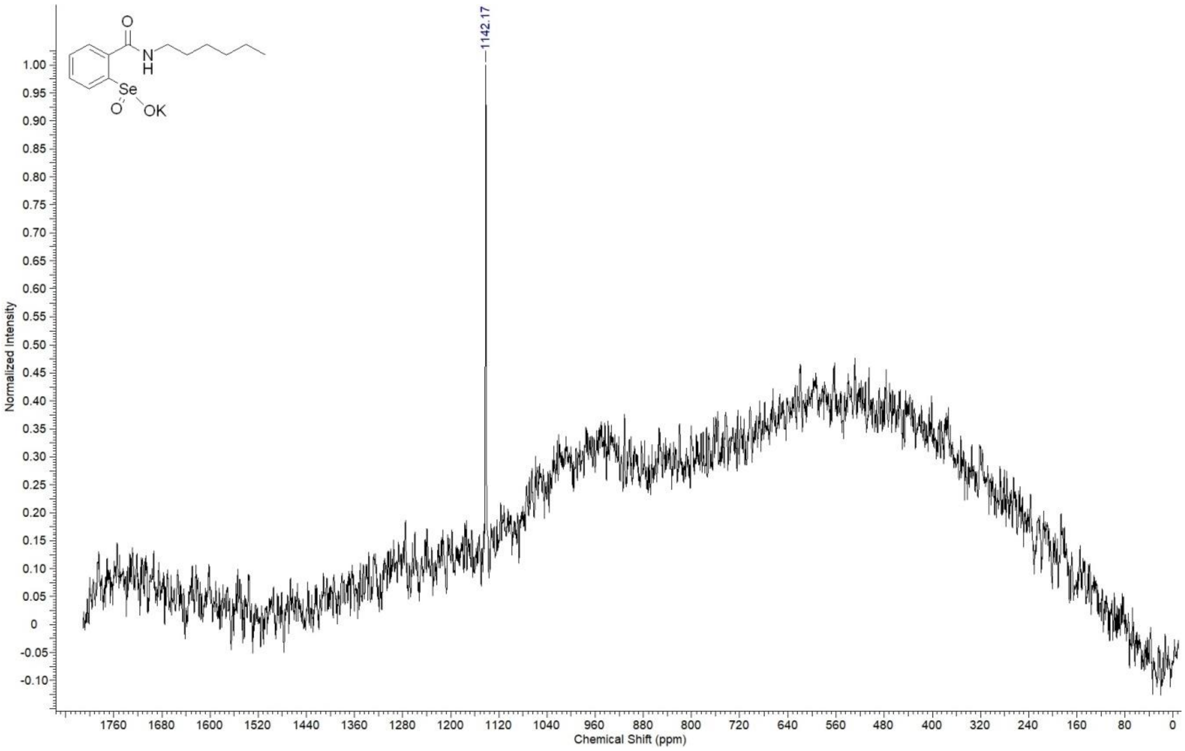

(c)

Figure S10. (**a**) ^{1}H NMR, (**b**) ^{13}C NMR, and (**c**) ^{77}Se NMR spectra of 22-(N-hexylcarboxyamido)-benzeneselenenic acid potassium salt 19.

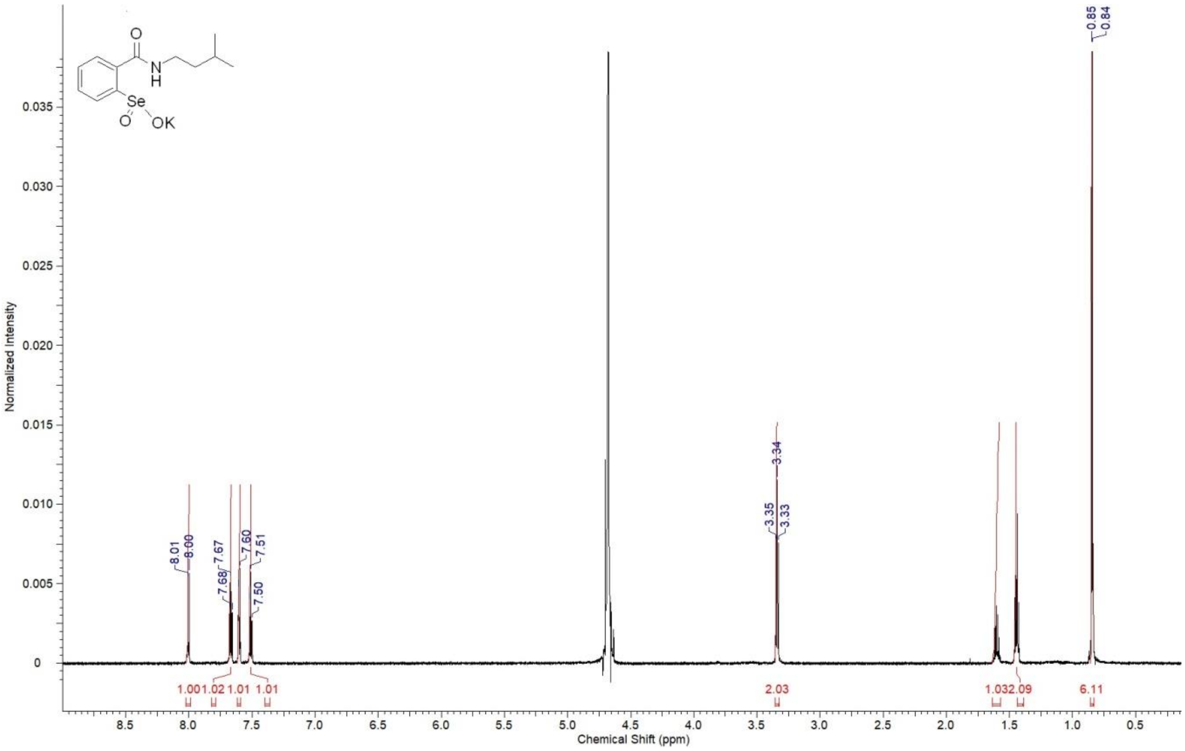

(a)

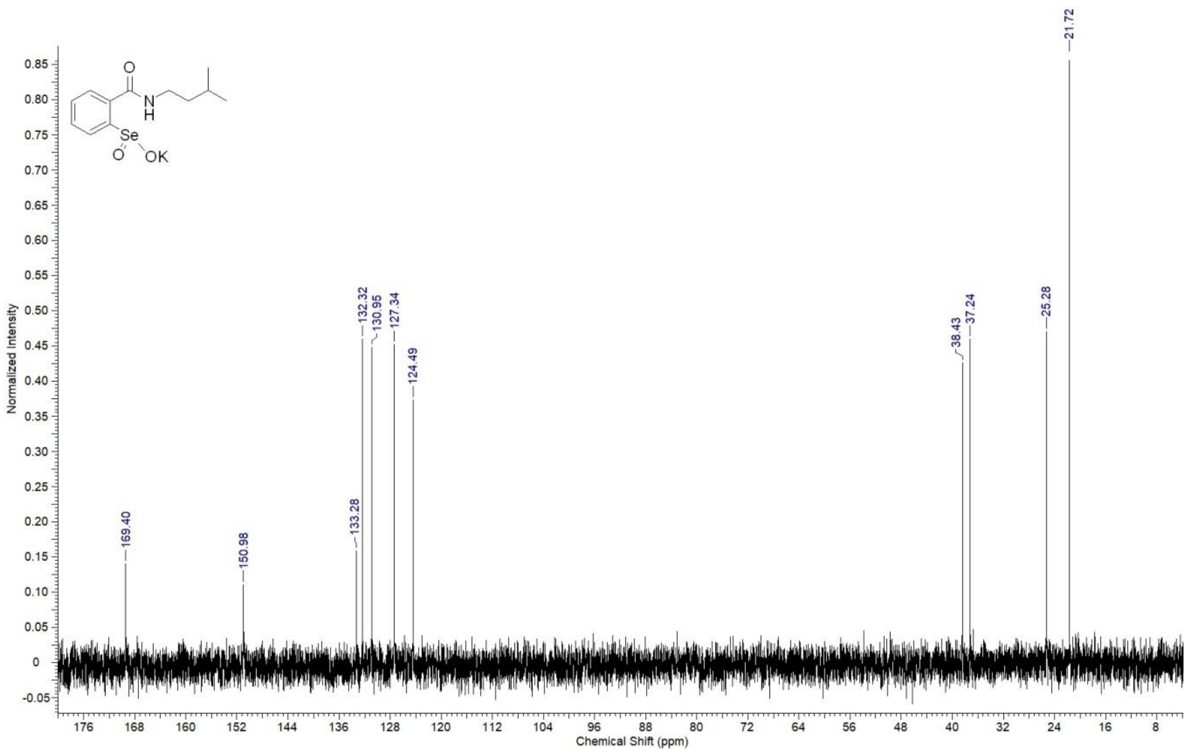

(b)

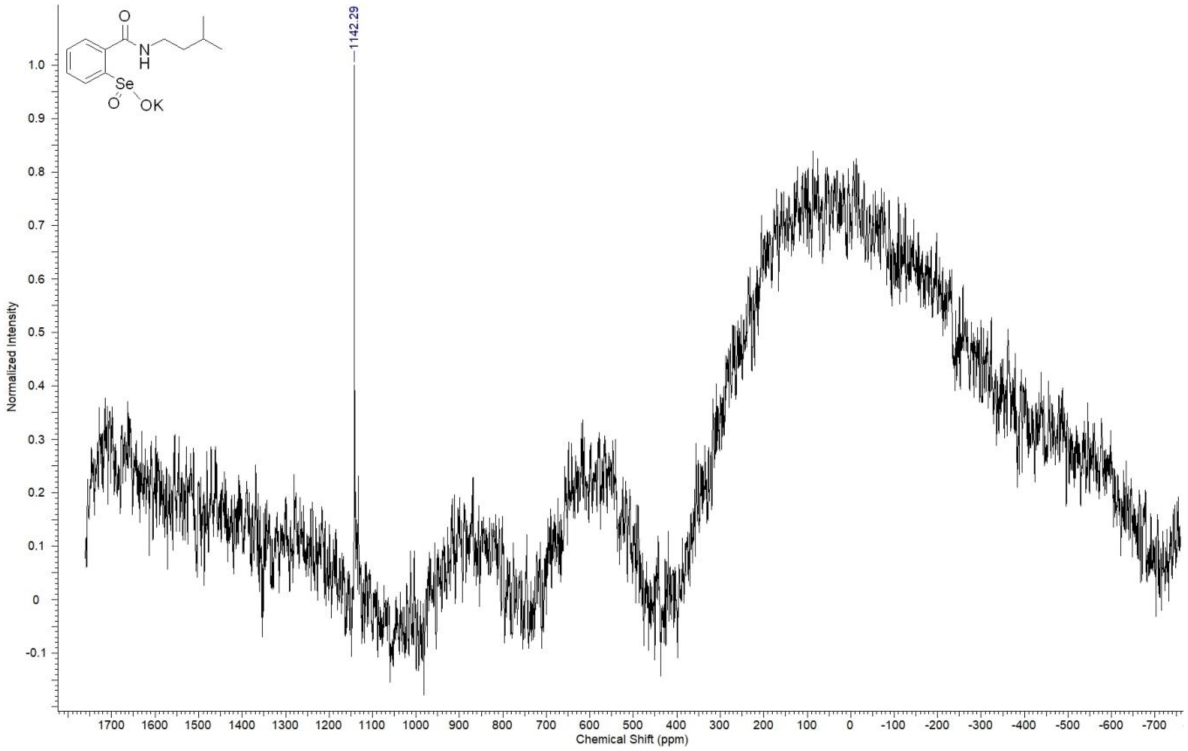

(c)

Figure S11. (**a**) ^{1}H NMR, (**b**) ^{13}C NMR, and (**c**) ^{77}Se NMR spectra of 2-(N-(3-methyl)butylcarboxy-amido)benzeneselenenic acid potassium salt 20.

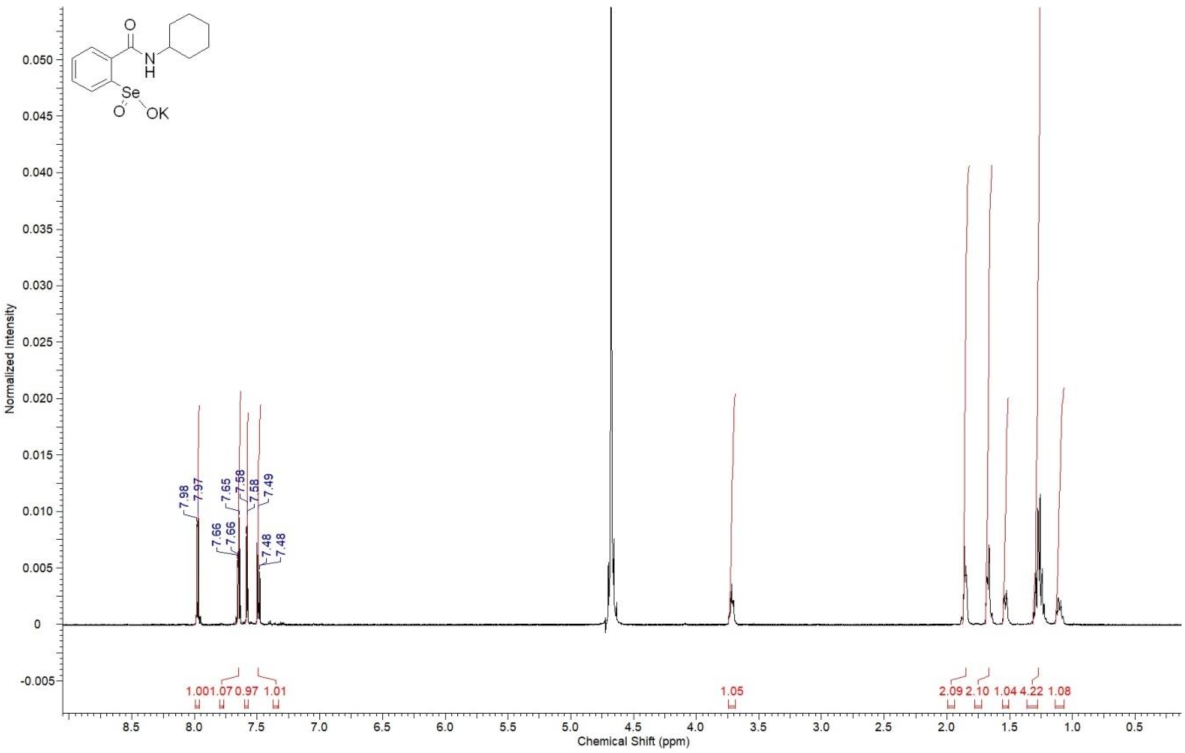
Se
OK
O
H
N
Normalized Intensity
Chemical Shift (ppm)
7.98
7.97
7.66
7.66
7.65
7.58
7.58
7.49
7.48
7.48
1.00
1.07
0.97
1.01
1.05
2.09
2.10
1.04
4.22
1.08

(a)

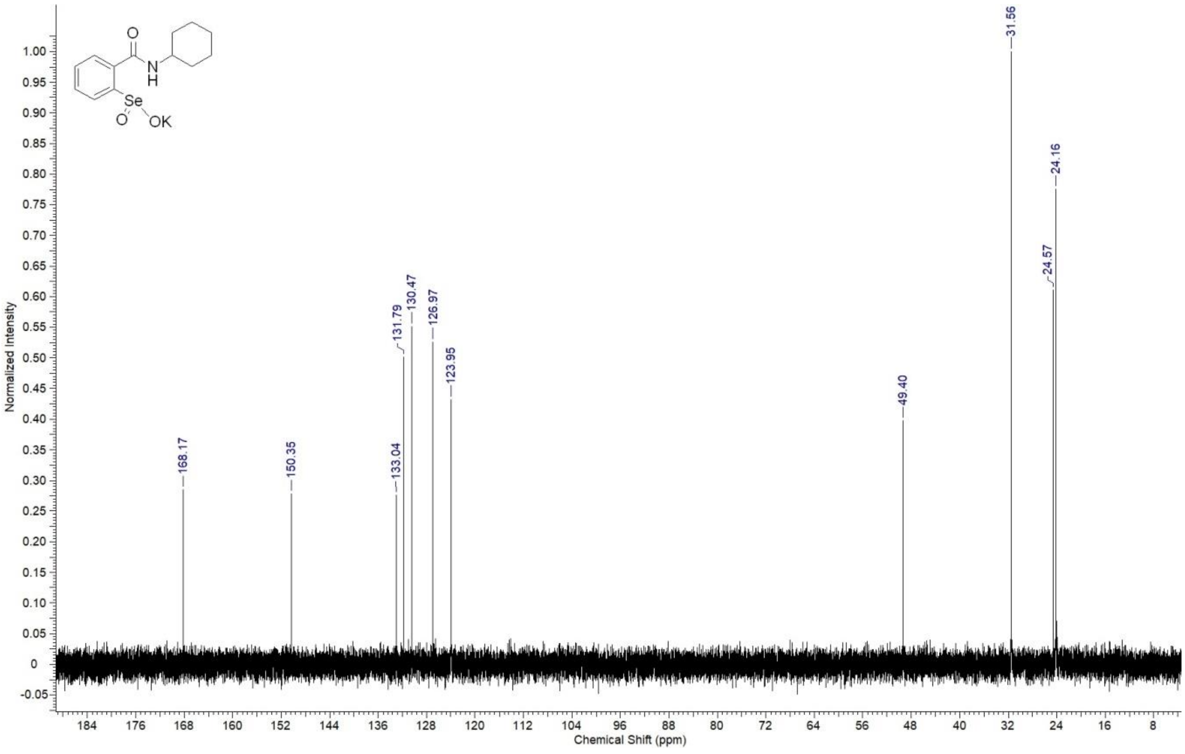
Se
OK
O
H
N
Normalized Intensity
Chemical Shift (ppm)
168.17
150.35
133.04
131.79
130.47
126.97
123.95
49.40
31.56
24.57
24.16

(b)

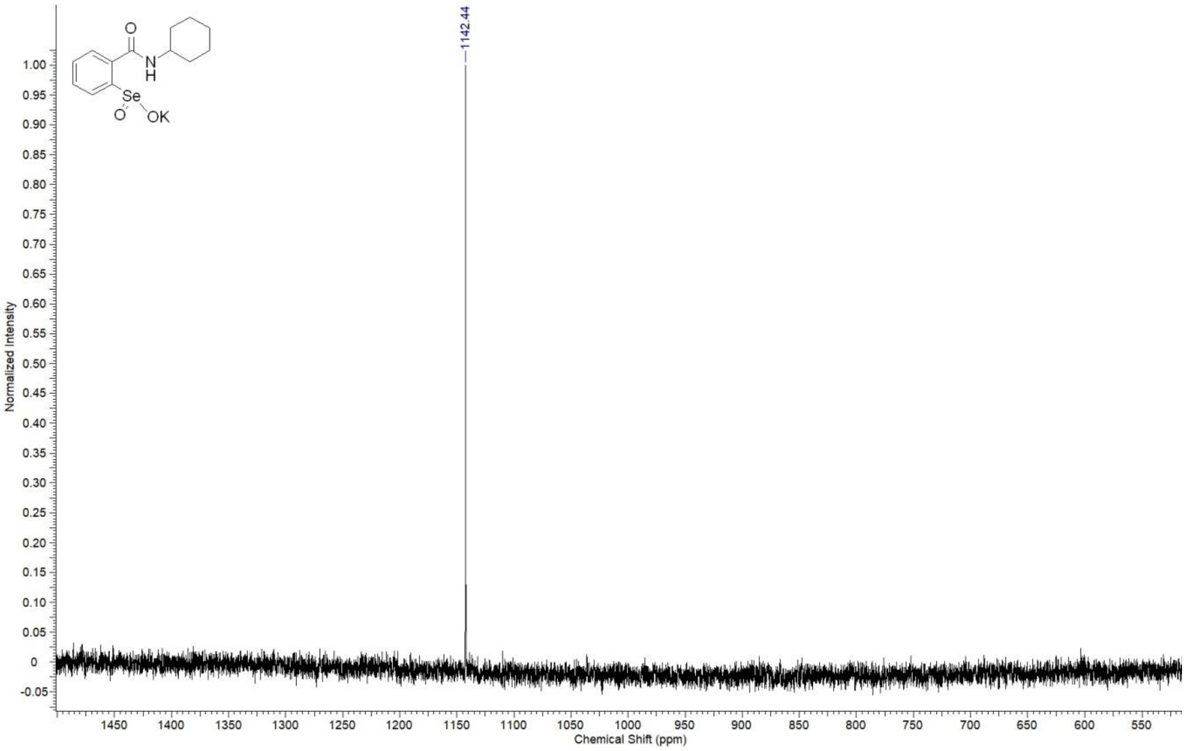

(c)

Figure S12. (**a**) ^{1}H NMR, (**b**) ^{13}C NMR, and (**c**) ^{77}Se NMR spectra of 2-(N-cyclohexylcarboxyamido)-benzeneselenenic acid potassium salt 21.

© 2020 by the authors. Submitted for possible open access publication under the terms and conditions of the Creative Commons Attribution (CC BY) license (http://creativecommons.org/licenses/by/4.0/).
